# Genome-wide association mapping for the identification of stripe rust resistance loci in US hard winter wheat

**DOI:** 10.1007/s00122-025-04858-3

**Published:** 2025-03-10

**Authors:** Rajat Sharma, Meinan Wang, Xianming Chen, Indira Priyadarshini Lakkakula, Paul St. Amand, Amy Bernardo, Guihua Bai, Robert L. Bowden, Brett F. Carver, Jeffrey D. Boehm, Meriem Aoun

**Affiliations:** 1 Department of Entomology and Plant Pathology, Oklahoma State University, Stillwater, OK USA; 2 Department of Plant Pathology, Washington State University, Pullman, WA USA; 3 USDA-ARS Wheat Health, Genetics, and Quality Research Unit, Pullman, WA USA; 4 USDA-ARS Hard Winter Wheat Genetics Research Unit, Manhattan, KS USA; 5 Department of Plant and Soil Sciences, Oklahoma State University, Stillwater, OK USA; 6 USDA-ARS Wheat, Sorghum & Forage Research Unit, Lincoln, NE USA

## Abstract

**Key message:**

The GWAS and testing with *Yr* gene linked markers identified 109 loci including 40 novel loci for all-stage and adult plant stage resistance to stripe rust in 459 US contemporary hard winter wheat genotypes.

**Abstract:**

Stripe rust is a destructive wheat disease, caused by *Puccinia striiformis* f. sp. *tritici* (*Pst*). To identify sources of stripe rust resistance in US contemporary hard winter wheat, a panel of 459 Great Plains wheat genotypes was evaluated at the seedling stage against five US *Pst* races and at the adult plant stage in field environments in Oklahoma, Kansas, and Washington. The results showed that 7–14% of the genotypes were resistant to *Pst* races at the seedling stage, whereas 32–78% of genotypes were resistant at the adult plant stage across field environments, indicating the presence of adult plant resistance. Sixteen genotypes displayed a broad spectrum of resistance to all five *Pst* races and across all field environments. The panel was further genotyped using 9858 single-nucleotide polymorphisms (SNPs) generated from multiplex restriction amplicon sequencing (MRASeq) and the functional DNA markers linked to the known stripe rust resistance (*Yr*) genes *Yr5*, *Yr15*, *Yr17*, *Yr18*, *Yr29*, *Yr36*, *Yr40*, *Yr46*, and *QYr.tamu-2B*. A genome-wide association study (GWAS) was performed using genotypic and phenotypic data, which identified 110 SNPs and the functional markers linked to *Yr15* and *Yr17* to be significantly associated with stripe rust response. In addition, *Yr5*, *Yr15*, *Yr17*, *Yr18*, *Yr29*, and *QYr.tamu-2B* were detected by their functional DNA markers in the panel. This study identified 40 novel loci associated with stripe rust resistance in genomic regions not previously characterized by known *Yr* genes. These findings offer significant opportunities to diversify and enhance stripe rust resistance in hard winter wheat.

**Supplementary Information:**

The online version contains supplementary material available at 10.1007/s00122-025-04858-3.

## Introduction

Wheat (*Triticum aestivum* L.) is an important staple food crop that provides calories for ~ 40% of the human population (Li et al. 2019). In the USA, wheat ranks third among field crops in terms of planted acreage, production, and gross farm receipts, trailing only corn and soybeans (USDA Economic Research Service 2024). Winter wheat constitutes approximately 70% of the total US wheat production, with hard winter wheat (HWW) being the most produced market class, which is primarily grown in the US Great Plains. Global wheat production is challenged by present and emerging biotic and abiotic stressors. One of which, wheat stripe rust, caused by the biotrophic fungus *Puccinia striiformis* f. sp. *tritici* (*Pst*), is an economically significant disease that has caused several major epidemics worldwide (Jamil et al. 2020). Stripe rust has been reported in over 60 countries across North America, South America, Asia, Africa, Australia, and Europe (Chen 2005), causing up to 100% yield losses depending on the cultivar planted, the timing of infection, and weather conditions. Stripe rust is favored by cool and humid climates. The pathogen has expanded its reach since the emergence of virulent *Pst* strains in the 2000s that were aggressive and adapted to warmer climates (Markell and Milus 2008; Milus et al. 2009; Chen et al. 2010). Approximately 88% of wheat-growing areas worldwide are now prone to stripe rust infection, including the US Great Plains where the disease has become more severe in HWW production zones (Wang et al. 2022). Global vulnerability to stripe rust is evidenced by annual losses estimated to exceed one billion US dollars (Beddow et al. 2015). In the USA, the Pacific Northwest (PNW) is a hotspot for stripe rust because of its conducive environment and high variability of *Pst* virulence to stripe rust resistance (*Yr*) genes present among planted cultivars. Due to increased production losses, an expanding geographic range into traditionally non-stripe rust areas, and the rapid virulence evolution of the pathogen, stripe rust is now considered the most damaging cereal rust (Milus et al. 2009; Wellings 2011; Goyal and Manoharachary 2014; Beddow et al. 2015). Breeding for wheat cultivars with durable stripe rust resistance is considered the most cost-effective and environmentally friendly strategy to manage this disease (Chen 2005).

Stripe rust resistance genes (*Yr*) can be classified into two types: all-stage resistance (ASR), also known as seedling resistance, and adult plant resistance (APR). ASR is effective during all growth stages and is typically characterized as having qualitative or monogenic resistance. ASR adheres to the gene-for-gene model described by Flor (1971), offering high levels of protection but is race-specific, and its effectiveness is compromised by high selection pressure on the pathogen, which may mutate to overcome resistance. In contrast, APR is more durable but often provides partial resistance. It is expressed or enhanced at the adult plant stage (Lagudah 2011; Chen 2013; Mundt 2014; Ellis et al. 2014). Moreover, APR is usually non-race specific, though race-specific APR has also been identified (Milus et al. 2015). Despite their durability, APR genes do not protect plants at the seedling stage and tend to show variability in the timing and levels of resistance across environments, where a single APR gene often provides insufficient protection under severe epidemics (Risk et al. 2012; Chen 2014; Singh et al. 2015). Pyramiding multiple APR genes is essential to provide a high level of resistance through additive or epistatic effects (Sørensen et al. 2014). To achieve a high level of resistance that is durable, breeders pyramid multiple effective ASR and APR genes or APR genes in a wheat cultivar are recommended.

To date, 86 *Yr* genes (*Yr1* to *Yr86*) with official designations have been identified, along with 77 genes with temporary names and more than 350 quantitative trait loci (QTL) (McIntosh et al. 2020; Zhu et al. 2023). Among the named *Yr* genes, 58 are ASR genes and 28 are APR genes. Most APR genes are temperature-sensitive and known as high-temperature adult plant (HTAP) resistance genes that are activated in warmer climates (Chen 2013; Chen and Line 1995a, b). Although HTAP resistance is partial, resistance conferred by these genes has not yet been compromised (Chen 2013). To date, only 11 *Yr* genes have been cloned, namely *Yr5*, *Yr7*, *Yr10* (*YrNAM*), *Yr15*, *Yr18*, *Yr27*, *Yr36*, *Yr46*, *YrSP*, *YrAS2388,* and *YrU1* (Fu et al. 2009; Krattinger et al. 2009; Liu et al. 2014; Moore et al. 2015; Marchal et al. 2018; Klymiuk et al. 2018; Zhang et al. 2019; Wang et al. 2020; Athiyannan et al. 2022; Ni et al. 2023), of which *Yr18*, *Yr36,* and *Yr46* are APR genes. Generally, ASR genes are associated with nucleotide-binding domain and leucine-rich repeat proteins (Sánchez-Martín and Keller 2021). These proteins recognize effector proteins produced by the pathogen to initiate effector-triggered immunity, thereby protecting the host (Gururani et al. 2012). In contrast, APR genes lack specific structural domains, feature more complex structures, and indirectly contribute to resistance (Sánchez-Martín and Keller 2021). For example, *Yr18* encodes an ATP-binding cassette (ABC) transporter, *Yr36* encodes a protein kinase (WKS1), and *Yr46* encodes a hexose transporter. Most ASR genes and some APR genes deployed in commercial wheat cultivars are no longer effective due to the emergence of virulent *Pst* races (Hovmøller et al. 2011; Sørensen et al. 2014; Wan and Chen 2014). Mu et al. (2020) reported that although *Yr9*, *Yr10*, *Yr17*, and *YrSP* are present in HWW breeding lines developed in the US Great Plains, the majority of those genes have already succumbed to emerging virulent *Pst* races. Hence, it is essential to assess the current vulnerability of elite Great Plains HWW breeding lines to stripe rust by mapping both ASR and APR genes present among elite breeding lines and characterizing potentially new sources of resistance which will enable breeders to strategically pyramid combinations of genes in new wheat cultivars.

Traditionally, genetic loci conferring resistance have been identified through biparental linkage mapping (Xu et al. 2017). However, linkage mapping is limited by the time associated with the development of bi-parental populations and poor mapping resolution due to low recombination events within such populations (Flint-Garcia 2013). Alternatively, genome-wide association studies (GWAS) have been utilized to detect stripe rust resistance loci in different market classes of wheat (Naruoka et al. 2015; Liu et al. 2018, 2020; Mu et al. 2020; Muleta et al. 2020; Aoun et al. 2021b, c; Zhang et al. 2021; Jambuthenne et al. 2022; El Messoadi et al. 2024; Gao et al. 2024; Qiao et al. 2024). However, no comprehensive association mapping studies have yet to investigate stripe rust resistance loci/genes in contemporary hard winter wheat (HWW) germplasm. GWAS offers two main advantages over linkage mapping: (1) It provides much finer mapping resolution, as it uses a diverse panel of genotypes that has more ancestral recombination events at the population level, and (2) it exploits the genetic diversity in natural populations, thereby saving time and resources that would otherwise be spent in developing biparental populations (Yu and Buckler 2006). However, the low detection power for rare alleles and spurious associations due to population structure is the limitation of using GWAS. Notwithstanding, GWAS benefits from the use of a large population size, high marker density that uniformly covers the entire genome, and a mixed linear model to accurately identify genotype–phenotype associations (Bulli et al. 2016).

Advances in next-generation sequencing (NGS) technologies have facilitated the widespread adoption of several high-density single-nucleotide polymorphism (SNP) arrays such as Illumina Wheat 9 K iSelect SNP array, Wheat 15 K SNP array, Wheat Breeders’ 35 K Axiom array, Illumina Wheat 90 K iSelect SNP genotyping array, Axiom® Wheat 660 K SNP array, Axiom® HD Wheat genotyping (820 K) array, and genotyping-by-sequencing (GBS) (Cavanagh et al. 2013; Wang et al. 2014; Boeven et al. 2016; Winfield et al. 2016; Allen et al. 2017; Liu et al. 2020). SNP genotyping arrays generally produce high-quality SNPs but are expensive for a large population size (Bassi et al. 2016). GBS, while relatively less expensive, is a proprietary technology with high license and royalty fees, which have limited its use to only a few public and private institutions (Bernardo et al. 2020). Recently, the Multiplex Restriction Amplicon Sequencing (MRASeq) platform has been established (Bernardo et al. 2020). This method employs a two-step PCR approach to construct a library of amplicons for SNP discovery and genotyping, and SNPs are uniformly distributed throughout the genome, making MRASeq a novel, low-cost, high-throughput genotyping platform for routine breeding applications.

Herein, we assembled a diverse panel consisting of 459 HWW breeding lines and cultivars developed for wheat production in the US Great Plains and evaluated them for ASR and APR resistance to stripe rust. We further performed GWAS to identify genomic regions associated with stripe rust resistance. The identified resistance sources in this study can be deployed in breeding programs to enhance stripe rust resistance. Moreover, the stripe rust resistance loci identified in this study can be integrated into breeding programs through marker-assisted selection. Such integration will enable the stacking of multiple ASR and APR genes to develop wheat cultivars with broad spectrum and durable stripe rust resistance, ultimately mitigating yield losses and reducing the risk posed by emerging *Pst* races.

## Materials and methods

### Plant materials

A panel consisting of 459 US HWW breeding lines and cultivars was used in this study (Supplementary Table S1). The panel was selected from the 2021 and 2022 HWW regional performance nurseries described by Lakkakula et al. (2025). These nurseries included: (1) Northern Regional Performance Nursery (NRPN), consisting of advanced breeding lines primarily developed for cultivation in the Northern Great Plains; (2) Southern Regional Performance Nursery (SRPN), consisting of advanced breeding lines developed for cultivation in the Southern US Great Plains, and (3) Regional Germplasm Observation Nursery (RGON), consisting of other experimental breeding lines developed in various breeding programs of the US Great Plains. These genotypes originated from the US Department of Agriculture–Agricultural Research Service (USDA-ARS, Lincoln, NE) and multiple public and private breeding programs across 13 US states in the Great Plains.

### Stripe rust evaluation at the seedling stage in the greenhouse

The genotypes were evaluated at the seedling stage against five *Pst* races in the USA, namely PSTv-4, PSTv-14, PSTv-37, PSTv-40, and PSTv-52 (Wan and Chen 2014; Wang et al. 2022). PSTv-37 is the most predominant and widely distributed race across the USA, and PSTv-52 has been the second most prevalent race in the Great Plains in recent years. The other races used for evaluation were collected in Washington state, where more diverse *Pst* races are present. The five races collectively cover all virulence factors identified so far in the US Information on the virulence/avirulence phenotypes of the five *Pst* races on differential wheat lines (each carrying a single *Yr* gene) at the seedling stage that are presented in Supplementary Table S2.

Five to six seeds per genotype, along with the susceptible check “Avocet S, ” were planted in 48-well flat trays filled with a soil mixture (6 gallons of peat moss, 2 gallons of perlite, 3 gallons of sand, 3 gallons of commercial potting soil (sunshine mix), 4 gallons of vermiculite, 250 g Osmocote 14–14-14, and 2 gallons of water). The genotypes were planted in an augmented design, with the susceptible check included once in each tray. Alongside each *Pst* race experiment, a set of 18 single *Yr* gene differential lines was also planted to confirm the race identity of the race used for inoculation. At the second leaf stage, which is approximately 10–12-day post-planting, seedlings were uniformly inoculated with a spore suspension of fresh urediniospores in Novec 7100, at a concentration of 10 mg mL^−1^. Each 48-well flat tray received 6 mL of spore suspension. The inoculated plants were kept in a dark dew chamber at a temperature of 10 °C and 100% relative humidity for 24 h. Subsequently, the trays were moved to a rust-free growth chamber with a diurnal temperature cycle that gradually changed from 4 °C at 2 AM to 20 °C at 2 PM and a 16-h photoperiod. Infection types (ITs) were recorded 18–20-days post-inoculation using a 0 to 9 scale (Line and Qayoum 1992; Wan et al. 2017). This scale categorizes infected plants based on visible symptoms and sporulation, where IT “0” indicates the absence of any sporulation and symptoms; “1” is assigned to necrotic and/or chlorotic flecks without sporulation; “2” shows necrotic and/or chlorotic blotches or stripes without sporulation; “3” represents similar symptoms to “2” but with trace sporulation; IT of “4,” “5,” and “6” are given for light, intermediate, and moderate sporulation on necrotic and/or chlorotic blotches or stripes, respectively, while ITs of “7,” “8,” and “9” show abundant sporulation with necrotic and/or chlorotic blotches or stripes, chlorosis behind the sporulating area, and no necrosis or chlorosis behind the sporulation area, respectively. Plants with IT from 0 to 3 were considered resistant, those with IT from 4 to 6 were considered intermediate (moderately resistant), and IT from 7 to 9 indicated susceptibility (Wan et al. 2017).

### Stripe rust evaluation at the adult plant stage in the field

The 459 genotypes were evaluated for stripe rust response at the adult plant stage across four field environments during the 2022–2023 seasons. The locations included Chickasha in Oklahoma, Rossville in Kansas, Pullman, and Mount Vernon in Washington state. These locations feature a wide geographic range and have different environmental conditions and compositions of *Pst* races. For example, Chickasha and Rossville typically experience relatively warmer and drier climates, whereas Pullman and Mount Vernon are generally characterized by cooler temperatures and Mount Vernon is relatively humid and mild compared to Pullman. To ensure an optimal level of infection, artificial inoculation with local races specific to each location was performed at Chickasha, Rossville, and Pullman, while at Mount Vernon, we relied on natural infection. Standard management practices were adopted to grow plants in the stripe rust evaluation nurseries.

The wheat genotypes were planted in the fall of 2022 in non-replicated rows using an augmented design. At Chickasha, “Pete” was planted as the susceptible check every 50 rows and used as spreader rows to ensure uniform and high levels of stripe rust infection. Stripe rust response was recorded on flag leaves for IT and disease severity (DS) at the Feekes stage 10.5–11.0 (Large 1954). However, ITs from Chickasha were difficult to rate due to a sudden temperature increase that led to the conversion of urediniospores into teliospores. At Pullman and Mount Vernon, “PS 279” was used as a susceptible check and was also planted around the experimental field as borders to aid the spread of urediniospores. At Pullman, IT and DS data were recorded three times at the ripening stage at three-day intervals when PS 279 showed more than 80% DS. At Mount Vernon, data were recorded at the jointing stage (PS 279 > 80% DS) and at the Feekes stage 10.5–11.0 (PS 279 > 95% DS). At Rossville, “Jagalene” was planted after every 80 rows as a susceptible check and as spreader rows. Data for IT and DS were recorded when Jagalene showed more than 80% DS, with an additional DS reading taken five days after the first reading. The area under the disease progress curve (AUDPC) was calculated from multiple DS readings from Pullman and two DS readings from Rossville according to the formula: AUDPC = ∑ [(*X*_*i*_ + *X*_*i*+*1*_)/2] *t*_*i*_, where *X*_*i*_ is DS value on date *i*, and *t*_*i*_ is days between dates *i* and *i* + 1. AUDPC was then converted to relative AUDPC (rAUDPC), in which the AUDPC of the susceptible check is treated as 100% and the other genotypes’ AUDPC values are converted to percentages of the susceptible check’s AUDPC (Chen and Line 1995a; Liu et al. 2020). Best linear unbiased estimates (BLUEs) for IT and DS were also extracted across all environments using a linear mixed model in the R package “lme4” (Vazquez et al. 2010; Bates et al. 2015), where genotype was considered as a fixed effect and environment was considered as a random effect (Table S1). Hereafter disease ratings in these field environments are designated as CH for Chickasha, RS 1 for the first rating in Rossville, RS 2 for the second rating in Rossville, PL 1 for the first rating in Pullman, PL 2 for the second rating in Pullman, PL 3 for the third rating in Pullman, MV 1 for the first rating in Mount Vernon, MV 2 for the second rating in Mount Vernon, and BLUE for best linear unbiased estimates across environments (Table 1).

**Table 1:** Mean stripe rust responses in different evaluation tests

Trait/environment^a^	Designation^b^	Mean ± SE^c^
PSTv-4 (seedling stage)	PSTv-4^s^	8.1 ± 0.08
PSTv-14 (seedling stage)	PSTv-14^s^	8.2 ± 0.08
PSTv-37 (seedling stage)	PSTv-37^s^	8.2 ± 0.08
PSTv-40 (seedling stage)	PSTv-40^s^	7.7 ± 0.08
PSTv-52 (seedling stage)	PSTv-52^s^	7.6 ± 0.09
PSTv-14 (adult plant stage)	PSTv-14^a^	3.1 ± 0.16
PSTv-37 (adult plant stage)	PSTv-37^a^	3.8 ± 0.16
PSTv-40 (adult plant stage)	PSTv-40^a^	2.1 ± 0.12
DS–Chickasha, OK (May 22, 2023)	CH (DS)	20.3 ± 0.91
IT–MV 1 = Mt. Vernon, WA (April 27, 2023)	MV 1 (IT)	4.5 ± 0.12
DS–MV 1 = Mt. Vernon, WA (April 27, 2023)	MV 1 (DS)	38.9 ± 1.12
IT–MV 2 = Mt. Vernon, WA (June 7, 2023)	MV 2 (IT)	5.7 ± 0.12
DS–MV 2 = Mt. Vernon, WA (June 7, 2023)	MV 2 (DS)	54.7 ± 1.38
IT–PL 1 = Pullman, WA (June 16, 2023)	PL 1 (IT)	5.5 ± 0.1
DS–PL 1 = Pullman, WA (June 16, 2023)	PL 1 (DS)	24.4 ± 0.75
IT–PL 2 = Pullman, WA (June 19, 2023)	PL 2 (IT)	4.3 ± 0.11
DS–PL 2 = Pullman, WA (June 19, 2023)	PL 2 (DS)	36.1 ± 1.2
IT–PL 3 = Pullman, WA (June 22, 2023)	PL 3 (IT)	5.2 ± 0.12
DS–PL 3 = Pullman, WA (June 22, 2023)	PL 3 (DS)	36 ± 1.14
Relative AUDPC–Pullman DS	PL rAUDPC	33.2 ± 1.03
IT–Rossville, KS (May 25, 2023)	RS 1 (IT)	2.3 ± 0.1
DS–Rossville, KS (May 25, 2023)	RS 1 (DS)	16 ± 0.93
DS–Rossville, KS (May 30, 2023)	RS 2 (DS)	29.2 ± 1.36
Relative AUDPC–Rossville DS	RS rAUDPC	22.3 ± 1.07
IT–Multi-environment BLUE	BLUE (IT)	4.6 ± 0.09
DS–Multi-environment BLUE	BLUE (DS)	32 ± 0.86

^a^IT = infection type; DS = disease severity; AUDPC = area under disease progress curve; BLUE = best linear unbiased estimates

^b^s = infection type at the seedling stage; a = infection type at the adult plant stage

### Genotyping, population structure, and linkage disequilibrium

The 459 HWW genotypes were genotyped using MRASeq (Bernardo et al. 2020) at the USDA-ARS Genotyping Lab in Manhattan, KS, as described by Lakkakula et al. (2025). SNP calling was performed using TASSEL software v.5 (Bradbury et al. 2007), and the physical positions of the SNPs were assigned based on the Chinese Spring reference genome RefSeq v2.1 developed by the International Wheat Genome Sequencing Consortium (IWGSC) (Zhu et al. 2021). Following the same data processing workflow as Lakkakula et al. (2025), SNPs with ≤ 65% missing data were retained for imputation using Beagle 5 (Browning et al. 2018), and markers with heterozygosity ≥ 15% or minor allele frequency (MAF) ≤ 5% were excluded from downstream analyses. This resulted in 9,858 high-quality SNPs, which were used for population structure and GWAS analyses. Additionally, the panel was genotyped for the following functional DNA markers linked to nine known *Yr* genes and quantitative trait loci (QTL): *Yr5*, *Yr15*, *Yr17*, *Yr18*, *Yr29*, *Yr36*, *Yr40*, *Yr46*, and *QYr.tamu-2B*. The physical positions of the characterized *Yr* genes */*QTL were based on Tong et al. (2024). Information on primer sequences and PCR protocols for these functional markers is available upon request from the USDA-ARS Genotyping Lab, Manhattan, KS. Genotypic results for these *Yr* genes/QTL are presented in Supplementary Table S1.

Principal component analysis (PCA) and linkage disequilibrium (LD) analyses were performed as described by Lakkakula et al. (2025). Briefly, population structure was assessed using principal component analysis (PCA) on 9,858 filtered SNPs, and linkage disequilibrium (LD) decay was analyzed using *r*^*2*^ values between pairs of SNPs plotted against physical distances. Critical *r*^*2*^ values were determined based on unlinked markers, and LD decay was estimated at the intersection of the LOESS curve with the critical *r*^*2*^ value.

### Genome-wide association mapping

To identify loci associated with stripe rust responses at both the seedling and adult plant stages, GWAS was performed using the filtered 9,858 SNPs and phenotypic data for different *Pst* races and in multiple field environments. Association mapping was implemented in GAPIT 3 (Genomic Association and Prediction Integrated Tool v3) in R software (Wang and Zhang 2021). We used three different GWAS models, including mixed linear model (MLM) (Yu et al. 2006), fixed and random model circulating probability unification (FarmCPU) (Liu et al. 2016), and Bayesian-information and linkage-disequilibrium iteratively nested keyway (BLINK) (Huang et al. 2019). The single-locus MLM is traditionally the most used model for GWAS. It uses population structure (Q matrix) and kinship or family relatedness (K matrix) to control spurious associations (Zhang et al. 2005; VanRaden 2008). However, this model was designed to test one marker at a time and is more likely to cause spurious associations (Wen et al. 2018). Multi-locus models like FarmCPU and BLINK are considered more efficient and reliable than single-locus models for mapping studies (Vikas et al. 2022). FarmCPU operates iteratively, using both fixed and random models, and incorporates significant SNPs as cofactors in each iteration to manage spurious associations without overfitting the model (Liu et al. 2016). BLINK is an improved version of FarmCPU, incorporating two significant modifications. First, BLINK does not assume a uniform distribution of causal genes across the genome. Secondly, it focuses on individual markers rather than groups of markers (bins) and excludes markers in linkage disequilibrium (LD) with the most significant marker. This implies that significant markers in GWAS tag unique loci that are not in LD. BLINK employs Bayesian information criterion (BIC) within a fixed effect framework to estimate maximum likelihood (Huang et al. 2019). Studies reported that the BLINK model outperformed other models of GAPIT 3 in terms of statistical power and computational efficiency, generating fewer false positives and identifying more true associations than FarmCPU (Huang et al. 2019; Wang and Zhang 2021).

To determine marker-trait associations (MTAs), the GWAS models incorporated the K matrix and the optimal number of principal components (PCs) in the Q matrix was selected based on quantile–quantile (Q-Q) plots that visualize the deviation of marker observed − log10 (*P*) from the expected − log10 (*P*) (Megerssa et al. 2020; Aoun et al. 2021a, 2022). The number of PCs tested in the Q matrix was limited to the first four PCs. Significant associations were determined using a threshold of false discovery rate (FDR) ≤ 0.05 (Benjamini and Hochberg 1995). Manhattan plots were generated using the R package “CMplot” (https://github.com/YinLiLin/R-CMplot) and “geom_point” function in the R package “ggplot2” (Wickham and Sievert 2009).

## Results

### Phenotypic data analyses

**Fig. 1:**
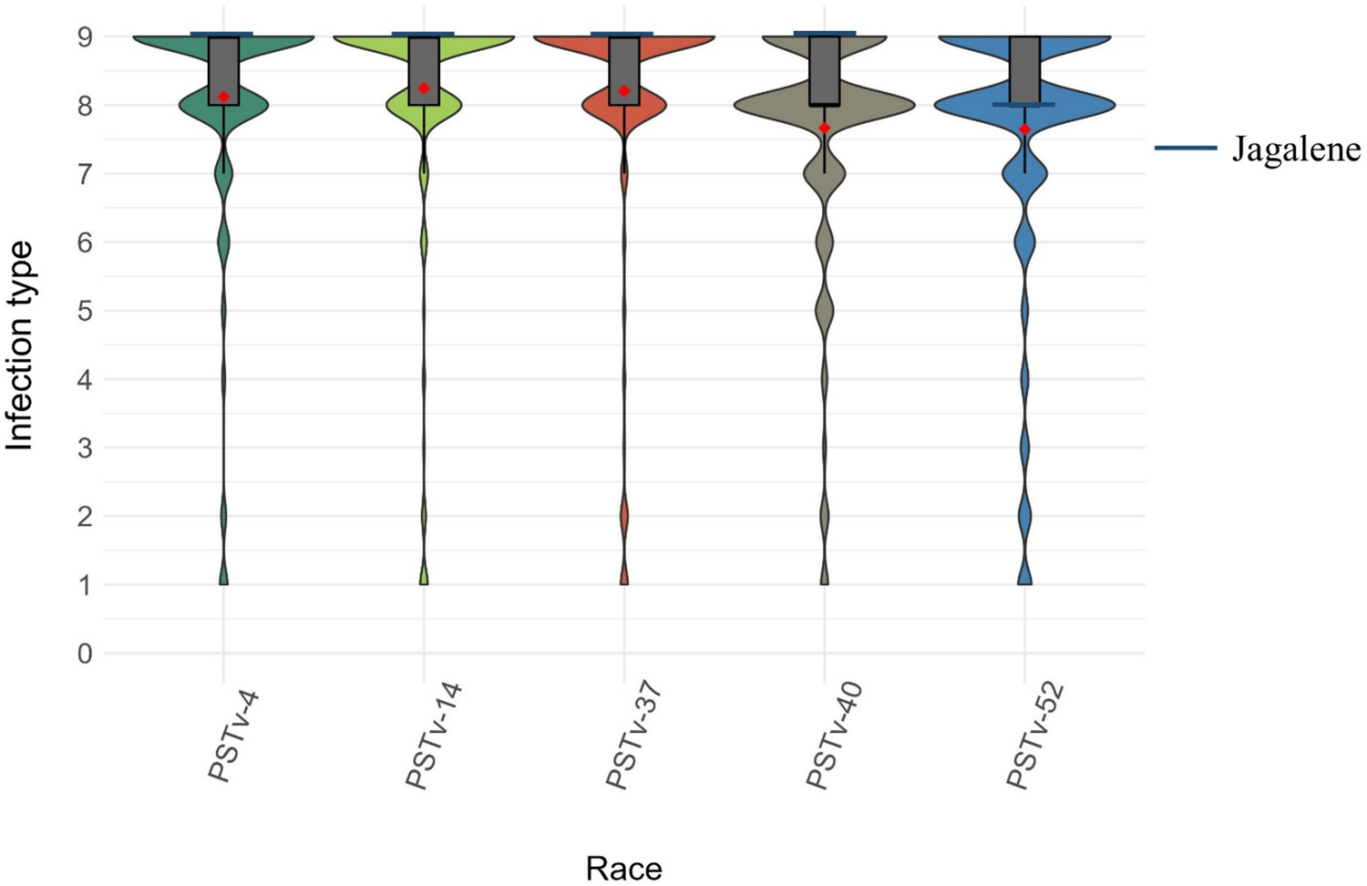
Distribution of infection types to five *Puccinia striiformis* f. sp. *tritici* races at the seedling stage in 459 hard winter wheat genotypes. The red diamonds represent the means. The blue horizontal lines correspond to stripe rust responses of the susceptible check Jagalene (colour figure online)

**Fig. 2:**
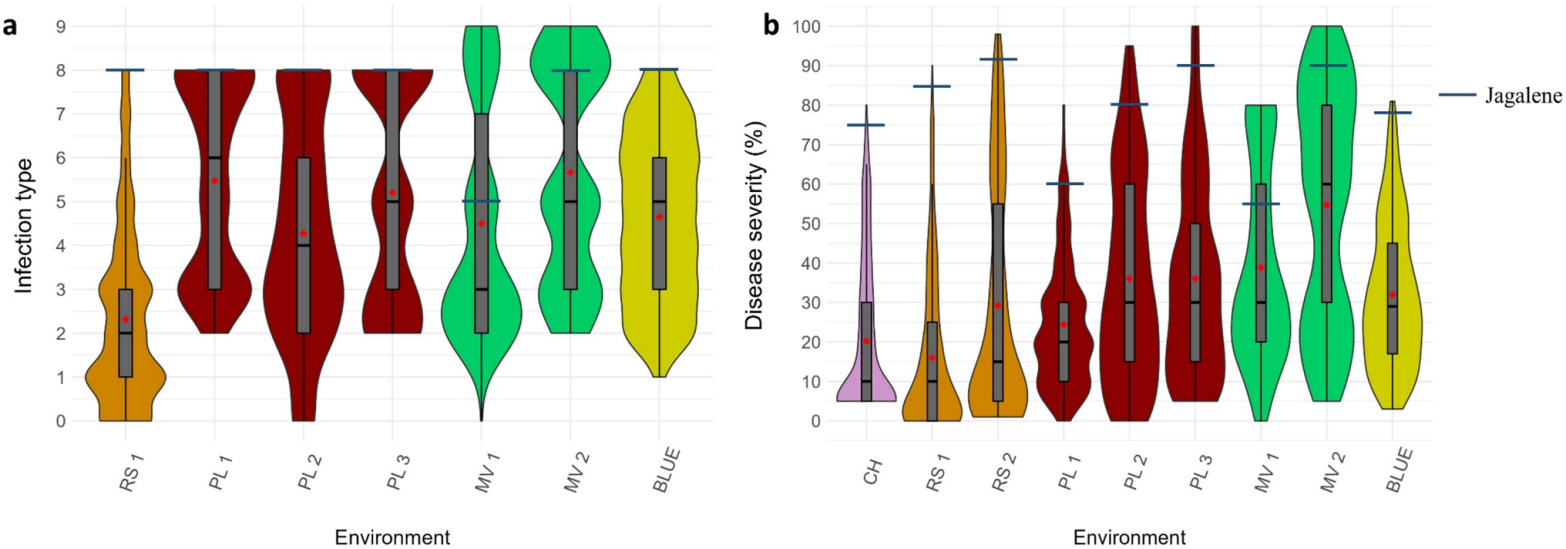
Distributions of stripe rust infection type (**a**) and disease severity (**b**) at the adult plant stage in 459 hard winter wheat genotypes across field environments. The black bold horizontal lines in the box plots denote the medians, and the red diamonds represent the means. The blue horizontal lines correspond to stripe rust responses of the susceptible check Jagalene. CH = Chickasha, OK; MV 1 = first disease rating at Mount Vernon, WA; MV 2 = second disease rating at Mount Vernon, WA; PL 1 = first disease rating at Pullman, WA; PL 2 = second disease rating at Pullman, WA; PL 3 = third disease rating at Pullman, WA; RS 1 = first disease rating at Rossville, KS; RS 2 = second disease rating at Rossville, KS; BLUE = multi-environment best linear unbiased estimates (colour figure online)

Descriptive statistical analysis and raw phenotypic data for IT at the seedling stage and for IT, DS, and rAUDPC at the adult plant stage are provided in Table 1, Supplementary Tables S1, S3, and S4. The mean seedling ITs against races PSTv-4, PSTv-14, PSTv-37, PSTv-40, and PSTv-52 were 8.1, 8.2, 8.2, 7.7, and 7.6, respectively (Table 1 and Supplementary Table S3). The distributions of seedling responses to the five *Pst* races showed skewness toward susceptibility (Fig. 1). For instance, 91%, 93%, 93%, 86%, and 86% of the genotypes in the panel were susceptible (IT 7–9) to races PSTv-4, PSTv-14, PSTv-37, PSTv-40, and PSTv-52, respectively (Supplementary Table S1). Moderate to high percentages of genotypes displayed resistance (IT 0–3) at the adult plant stage, ranging from 32% in MV 2 to 78% in RS 1 (Fig. 2). The frequency distribution of stripe rust response at the adult plant stage was skewed toward resistance in Chickasha (CH) and Rossville (RS 1 and RS 2). The mean IT ranged from 2.3 in RS 1 to 5.7 in MV 2 (Table 1). Disease severity (DS) mirrored IT results at the adult plant stage, with means ranging from 16% in RS 1 to 55% in MV 2. The rAUDPC ranged from 0.5 to 94 in Rossville, with a mean of 22.3, and from 1.25 to 92.5 in Pullman, with a mean of 33.2 (Supplementary Tables S1, S3 and Supplementary Fig. S1). Multi-environment BLUE across field environments at the adult plant stage showed mean IT and DS values of 4.6 and 32%, respectively (Supplementary Table S3). The higher levels of resistance were observed at the adult plant stage compared to that at the seedling stage. Furthermore, higher resistance was observed in Rossville and Chickasha than in Mount Vernon and Pullman.

Among the evaluated genotypes, 16 were resistant to all five tested *Pst* races at the seedling stage and across field environments at the adult plant stage (Table 2). These 16 genotypes, sourced from the RGON 2022, were from the wheat breeding programs in Colorado (n = 1), Kansas (n = 10), and Texas (*n* = 5). Based on DNA marker data, broad-spectrum ASR in most of these 16 genotypes (except CO19D304R) was associated with the presence of *Yr5*, *Yr15*, and *QYr.tamu-2B* (Table 2). The resistance in CO19D304R was not associated with any of the known *Yr* genes tested with the functional DNA markers.

**Table 2:** List of genotypes showing a broad spectrum of resistance to five *Puccinia striiformis f. sp. tritici* races and across field environments along with known stripe rust resistance (*Yr*) genes in each genotype based on molecular markers. These genotypes originated from RGON 2022

Genotype	Infection types at the seedling stage (0–9 scale)					Stripe rust response at the adult plant stage^a^									Molecular marker data^b^
	PSTv-4	PSTv-14	PSTv-37	PSTv-40	PSTv-52	CH (DS)	MV 2 (IT)	MV 2 (DS)	PL3 (IT)	PL3 (DS)	RS 1 (IT)	RS 1 (DS)	BLUE (IT)	BLUE (DS)	*Yr* genes
CO19D304R	1	1	2	2	1	5	2	5	2	5	0	0	1	3	Unknown
KS21HD144	2	2	2	2	1	5	2	10	2	5	1	5	2	7	*Yr15, Yr17, Yr29*
KS21HD147	2	2	2	2	2	5	3	20	2	10	1	0	2	10	*Yr5, Het-Yr17 and Yr29*
KS21HD154	2	2	4	2	2	5	2	10	2	10	1	0	2	10	*Het-Yr5, Yr17, Yr29 and QYr.tamu-2B*
KS21U7266-E1-B2	1	1	1	1	1	5	2	5	2	5	0	0	2	3	*Het-Yr15, Yr17, Het-Yr18, Het-Yr29 and QYr.tamu-2B*
KS21U7274-A-G149	4	2	3	2	2	5	3	10	2	5	0	0	2	3	*Het-Yr15, Yr17 and Yr29*
KS21U7321-B2-B7	1	1	1	1	1	30	2	5	2	5	0	0	1	6	*Yr5, Yr15, Yr17 and QYr.tamu-2B*
KS21U7445-H9-C3	1	1	1	1	1	40	2	5	2	10	0	0	2	10	*Yr5, Yr15, Yr17 and QYr.tamu-2B*
KS21U7494-G14-C6	1	1	1	1	1	20	2	5	2	5	0	0	1	5	*Yr5, Yr15, Yr29 and QYr.tamu-2B*
KS21U7494-H1-B8	1	1	1	1	1	5	2	5	2	5	0	0	1	3	*Yr5, Yr15, Yr29 and QYr.tamu-2B*
KS21U7494-H5-C1	1	1	2	1	1	5	2	5	2	5	0	0	1	4	*Yr5, Het-Yr15 and QYr.tamu-2B*
TX18DH266	1	1	2	2	2	5	3	10	2	5	0	0	2	7	*Yr29 and QYr.tamu-2B*
TX18DH303	1	1	2	2	2	5	3	10	2	5	0	0	2	5	*Yr5, Yr17, Yr29 and QYr.tamu-2B*
TX18DH305	1	1	1	1	1	5	3	10	2	5	0	0	2	5	*Yr5, Yr15, Yr17 and QYr.tamu-2B*
TX18DH313	1	1	2	2	2	5	3	10	2	5	0	0	2	6	*Yr5 and QYr.tamu-2B*
TX18DH319	2	2	2	2	2	5	3	30	2	10	3	5	3	11	*Yr15, Yr17 and Yr29*

^a^IT = infection type; DS = disease severity (%); CH = Chickasha, OK; WA; MV 2 = second disease rating at Mount Vernon, WA; PL 3 = third disease rating at Pullman; RS 1 = first disease rating at Rossville, KS; BLUE = multi-environment best linear unbiased estimates

^b^Het = heterozygous for a given gene based on molecular markers

Stripe rust evaluation at the adult plant stage of the NRPN and SRPN (n = 151) genotypes against *Pst* races (PSTv-14, PSTv-37, and PSTv-40) in the greenhouse revealed a skewed distribution toward resistance, with mean IT values ranging from 2.1 to 3.8 (Table 1, Supplementary Table S4 and Supplementary Fig. S2). PSTv-40 was the least virulent race to the NRPN and SRPN genotypes at the adult plant stage compared to the other two races PSTv-14 and PSTv-37. Furthermore, among the 459 HWW genotypes, 59 genotypes were susceptible to the five tested *Pst* races at the seedling stage but showing resistant responses at the adult plant stage across various field environments (Supplementary Table S5). Thus, these genotypes likely carry exclusively APR genes.

Significant positive correlations were observed for IT (*r* = 0.61–0.84) among the five *Pst* races tested at the seedling stage (Supplementary Table S6). For field environments, IT and DS values calculated using multi-environment BLUE showed high correlations (*r* = 0.67–0.93) among most environments except for CH and MV 1 which exhibited moderate but significant correlations (*r* = 0.51–0.63). Relatively weaker correlations (*r* = 0.07–0.40) were observed between stripe rust responses at the seedling stage and at the adult plant stage, indicating the potential impact of APR in the panel.

### Genotyping, population structure, and linkage disequilibrium

**Fig. 3:**
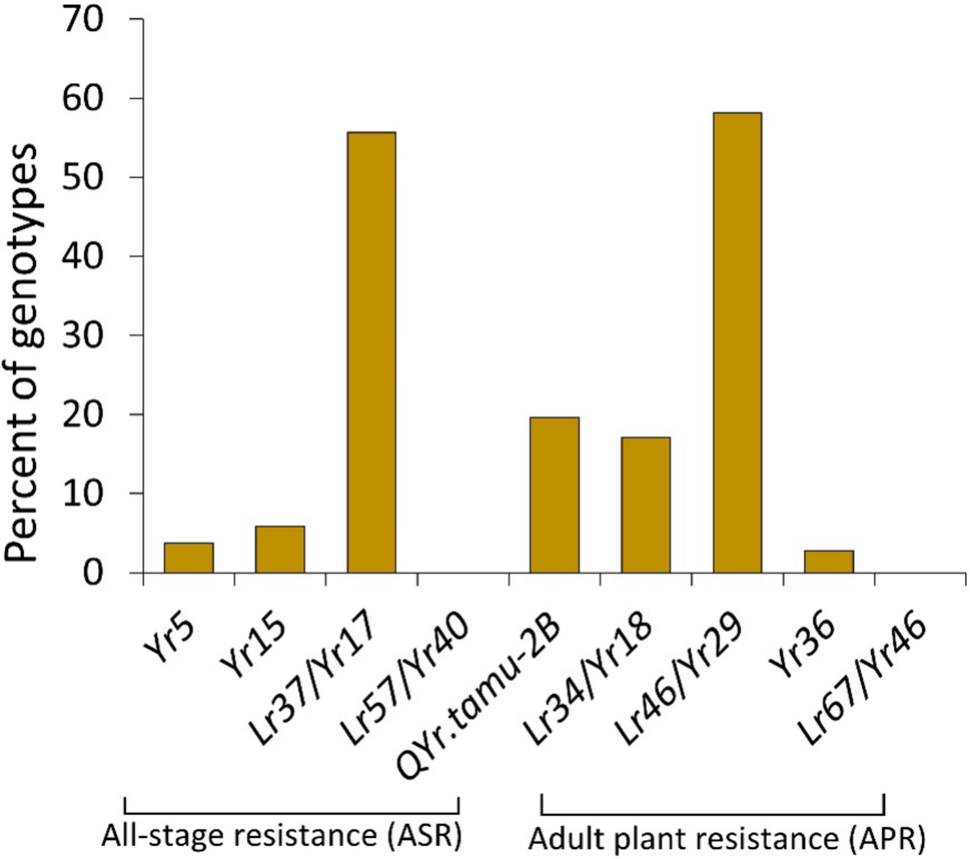
Frequencies of stripe rust resistance (*Yr*) genes in 459 hard winter wheat genotypes based on diagnostic molecular markers

Among the 9858 SNPs used for further analyses, 5227 SNPs (53.0%) were located on the A genome, 2336 (23.7%) on the B genome, and 2204 (22.4%) on the D genome, with 91 SNPs that were unaligned (UN) to a chromosome as described by Lakkakula et al. (2025). Chromosome 2A had the highest density of SNPs (*n* = 1005), followed by chromosome 4A (*n* = 924), whereas chromosome 4B had the lowest count at 225 SNPs. Based on functional DNA markers, the ASR genes *Yr17* and *QYr.tamu-2B* and the APR genes *Yr18* and *Yr29* were present at moderate to high frequencies (17–58%) in this HWW panel (Fig. 3). The broad-spectrum ASR genes *Yr5* and *Yr15*, to which no *Pst* race in the USA is virulent, had low frequencies of 4% and 6%, respectively. Only seven genotypes were found to carry both *Yr5* and *Yr15*: KS21U7321-B2-B7, KS21U7445-H9-C3, KS21U7445-H9-C6, KS21U7494-G14-C6, KS21U7494-H1-B8, KS21U7494-H5-C1, and TX18DH305, all of which were sourced from the RGON 2022. The HTAP resistance gene *Yr36* was found at a low frequency (3%). Furthermore, the ASR gene *Yr40* and the APR gene *Yr46* were not detected in the panel.

PCA showed a weak structure in this panel (Lakkakula et al. 2025). This suggests that Great Plains HWW genotypes are generally related and share ancestry with many common founder lines expected among their pedigrees. The first 10 PCs amounted for only 12.3% of the cumulative variation with the first four PCs explaining 2.7, 2.3, 1.4, and 1.2% of the variation, respectively. PCA grouped the genotypes into three clusters that are not associated with geographic regions. The genome-wide LD dropped to *r*^*2*^ of 0.1 within 0.8 Mb on average (Lakkakula et al. 2025). Furthermore, LD decayed to *r*^*2*^ threshold of 0.1 at approximately 0.9 Mb on average for the A genome, at 0.7 Mb on average for the B genome, and at 0.6 Mb on average for the D genome. Thus, SNPs within the aforementioned LD decay ranges or with an *r*^*2*^ value between SNPs greater than 0.1 were considered to represent the same QTL.

### GWAS model selection

The selection of the most suitable model for each trait was based on the examination of Q-Q plots (Supplementary Fig. S3). For most traits, BLINK model showed a sharp deviation of the observed *P*-value distribution from the expected, compared to those generated by FarmCPU and MLM models. These findings suggest that BLINK detected fewer false positives. Consequently, this study primarily discusses the marker-trait associations (MTAs) identified through BLINK (Tables 3 and 4), whereas GWAS results obtained from FarmCPU and MLM are provided in Supplementary Table S7. Nonetheless, significant associations identified by other models will also be considered, as validation by multiple models can further enhance the reliability of the associations found in this study. For all traits, the K matrix was included in the GWAS models and the optimal number of PCs in the Q matrix varied based on the trait. For instance, the BLINK models for traits PSTv-4^s^, PSTv-14^s^, PSTv-37^s^, PSTv-40^s^, MV 1 (IT), MV 1 (DS), PL 1 (DS), PL 2 (DS), PL 3 (IT), and RS 2 (DS) included no PCs (Q matrix was not included), whereas for traits PSTv-14^a^, RS 1 (IT), RS 1 (DS), and BLUE (IT), the first two PCs were included in the Q matrix. For traits PSTv-52^s^, PSTv-37^a^, CH (DS), MV 2 (IT), PL 1 (IT), PL 3 (DS), PL rAUDPC, and BLUE (DS), the first three PCs were included, whereas for traits MV 2 (DS), PL 2 (IT), and RS rAUDPC, the first four PCs were included in the Q matrix of the BLINK model.

**Table 3:** Summary of significant SNPs associated with stripe rust response at the seedling stage using the BLINK model

SNP^a^	Trait^b^	Chr.^c^	Position (bp)^d^	Alleles^e^	*P* value^f^	MAF^g^	FDR^h^	Effect	Markers identified using other GWAS models	Possible *Yr* gene
*S1A_332277563*	PSTv-52^s^	1A	332,277,563	G/**A**	9.52E-07	0.09	1.17E-03	− 0.52	FarmCPU	
*S1A_528048727*	PSTv-14^s^	1A	528,048,727	G/**A**	2.05E-08	0.07	5.06E-05	− 0.53		
*S1B_7915711*	PSTv-52^s^	1B	7,915,711	T/**C**	1.01E-05	0.15	9.03E-03	− 0.36		*Yr10*, *Yr84*
*S1B_7245353*	CH (DS)	1B	7,245,353	**G**/A	6.66E-06	0.07	8.21E-03	6.3		*Yr10*, *Yr84*
S1B_34102555*	PSTv-4^s^	1B	34,102,555	A/**G**	1.52E-08	0.06	7.48E-05	− 0.78	FarmCPU + MLM	*Yr85*
	BLUE (DS)	1B	34,102,555	A/**G**	3.29E-08	0.06	1.35E-04	− 7.91		*Yr85*
Yr15*	PSTv-4 s	1B	73,459,000	*Non-Yr15*/***Yr15***	4.10E-05	0.06	5.08E-02	− 0.59	MLM	*Yr15*
	PSTv-40 s	1B	73,459,000	*Non-Yr15*/***Yr15***	4.28E-08	0.06	1.41E-04	− 1.12	MLM	*Yr15*
S1B_141655194*	PSTv-4^s^	1B	141,655,194	T/**G**	5.49E-06	0.05	1.35E-02	− 0.63	MLM	
	PSTv-14^s^	1B	141,655,194	T/**G**	3.12E-13	0.05	1.54E-09	− 0.87	FarmCPU + MLM	
	PSTv-37^s^	1B	141,655,194	T/**G**	3.76E-15	0.05	3.71E-11	− 0.99	FarmCPU + MLM	
	PSTv-40^s^	1B	141,655,194	T/**G**	1.84E-13	0.05	1.82E-09	− 1.03	FarmCPU + MLM	
	PSTv-52^s^	1B	141,655,194	T/**G**	1.52E-18	0.05	1.50E-14	− 1.36	FarmCPU + MLM	
	MV 1 (DS)	1B	141,655,194	T/**G**	1.30E-05	0.05	2.14E-02	− 8.22	FarmCPU	
*S1B_308703553*	PSTv-14^s^	1B	308,703,553	G/**C**	2.07E-09	0.1	6.80E-06	− 0.51	FarmCPU + MLM	*Yr26*
*S1B_613082642*	PSTv-37^s^	1B	613,082,642	**A**/G	1.90E-06	0.07	3.74E-03	0.48		
***S2A_362922651***	PSTv-52^s^	2A	362,922,651	**C**/A	8.46E-06	0.14	8.49E-03	0.4		
***S2A_367020239***	PSTv-52^s^	2A	367,020,239	**C**/T	8.63E-07	0.16	1.17E-03	1.48		
*S2A_592670282*	PSTv-4^s^	2A	592,670,282	T/**C**	2.93E-05	0.05	4.66E-02	− 0.49		
*S2A_649238188*	PSTv-52^s^	2A	649,238,188	A/**G**	9.17E-07	0.06	1.17E-03	− 0.68	FarmCPU	
*S2A_655524120*	PSTv-52^s^	2A	655,524,120	T/**G**	2.41E-08	0.05	5.93E-05	− 0.79	FarmCPU + MLM	
*S2B_458606839*	PSTv-37^s^	2B	458,606,839	A/**G**	1.60E-10	0.08	7.88E-07	− 0.65	FarmCPU + MLM	
	PSTv-40^s^	2B	458,606,839	A/**G**	4.34E-09	0.08	2.14E-05	− 0.67	MLM	
	PSTv-52^s^	2B	458,606,839	A/**G**	6.61E-05	0.08	4.66E-02	− 0.43		
*S2B_523341745*	PSTv-52^s^	2B	523,341,745	C/**G**	8.59E-07	0.11	1.17E-03	− 0.48		
*S2B_598744752*	PSTv-4^s^	2B	598,744,752	T/**C**	4.49E-10	0.05	4.43E-06	− 1.06	FarmCPU + MLM	*Yr53*
	PSTv-14^s^	2B	598,744,752	T/**C**	1.57E-13	0.05	1.54E-09	− 1.23	FarmCPU + MLM	*Yr53*
	PSTv-37^s^	2B	598,744,752	T/**C**	2.38E-07	0.05	5.86E-04	− 0.88	FarmCPU + MLM	*Yr53*
	PSTv-52^s^	2B	598,744,752	T/**C**	1.62E-05	0.05	1.23E-02	− 0.76	MLM	*Yr53*
*S2B_706506028*	PSTv-14^s^	2B	706,506,028	T/**C**	2.22E-06	0.09	3.12E-03	− 0.59	MLM	*Yr5*
	PSTv-37^s^	2B	706,506,028	T/**C**	1.53E-05	0.09	1.88E-02	− 0.46	MLM	*Yr5*
	PSTv-40^s^	2B	706,506,028	T/**C**	2.97E-06	0.09	7.31E-03	− 0.55	MLM	*Yr5*
	PSTv-52^s^	2B	706,506,028	T/**C**	1.41E-05	0.09	1.16E-02	− 0.54	MLM	*Yr5*
*S2B_710041043*	PSTv-14^s^	2B	710,041,043	**G**/T	3.33E-07	0.07	5.47E-04	0.58		*Yr5*
S2B_724829085*	PSTv-14^s^	2B	724,829,085	T/**A**	2.50E-07	0.1	4.92E-04	− 0.43	MLM	
	PSTv-37^s^	2B	724,829,085	T/**A**	2.34E-07	0.1	5.86E-04	− 0.47	FarmCPU + MLM	
	PSTv-40^s^	2B	724,829,085	T/**A**	1.41E-06	0.1	4.63E-03	− 0.47	FarmCPU + MLM	
	MV 2 (DS)	2B	724,829,085	T/**A**	7.00E-06	0.1	1.38E-02	− 7.75	FarmCPU	
***S4A_61065066***	PSTv-4^s^	4A	61,065,066	**G**/T	4.06E-05	0.05	5.01E-02	0.52		
	PSTv-37^s^	4A	61,065,066	**G**/T	2.99E-06	0.05	4.91E-03	0.58	FarmCPU	
*S4A_311417587*	PSTv-14^s^	4A	311,417,587	T/**A**	5.42E-05	0.06	4.86E-02	− 0.4	FarmCPU	
***S4A_337252413***	PSTv-37^s^	4A	337,252,413	T/**G**	4.11E-06	0.08	5.79E-03	− 0.46	FarmCPU	
***S5A_392096163***	PSTv-4^s^	5A	392,096,163	G/**A**	1.40E-05	0.07	2.77E-02	− 0.44	FarmCPU	
*S5B_408312043*	PSTv-14^s^	5B	408,312,043	T/**A**	1.24E-05	0.06	1.22E-02	− 0.42	FarmCPU	
*S6A_92638652*	PSTv-14^s^	6A	92,638,652	G/**A**	7.36E-06	0.09	8.06E-03	− 0.35		
*S6A_498220952*	PSTv-4^s^	6A	498,220,952	T/**A**	3.31E-05	0.05	4.66E-02	− 0.52	FarmCPU + MLM	
*S6B_223467058*	PSTv-4^s^	6B	223,467,058	G/**T**	2.56E-07	0.14	8.43E-04	− 0.4	FarmCPU	
*S6D_74284236*	PSTv-52^s^	6D	74,284,236	G/**T**	2.72E-09	0.15	1.34E-05	− 0.56	FarmCPU	
*S7A_278687032*	PSTv-14^s^	7A	278,687,032	**G**/A	2.92E-06	0.35	3.59E-03	0.22	FarmCPU	
***S7A_339701189***	PSTv-52^s^	7A	339,701,189	**G**/C	8.62E-06	0.06	8.49E-03	0.59	FarmCPU	
***S7B_206209604***	PSTv-52^s^	7B	206,209,604	**G**/A	4.62E-09	0.05	1.52E-05	0.88		

^a^Underlined SNPs represent same QTL based on linkage disequilibrium (LD) between significant SNP marker pairs (r^2^ ≥ 0.1); ^*^ = SNPs associated with both seedling and adult plant stage; the SNPs in bold are potentially novel SNPs based on comparison with previously known *Yr* genes and QTL

^b^Trait code (s = infection type at the seedling stage, a = infection type at adult plant stage)

^c^Chromosome

^d^Physical positions of SNPs were based on Wheat Chinese Spring IWGSC RefSeq v2.1 (Zhu et al. 2021). Physical positions of characterized *Yr* genes/QTL were based on Tong et al. (2024)

^e^SNP major allele/minor allele, the allele in bold is associated with stripe rust resistance

^f^*P* value of the significant SNP

^g^Minor allele frequency of the significant SNP

^h^False discovery rate of the significant SNP

**Table 4:** Summary of significant SNPs associated with stripe rust response at the adult plant stage using BLINK model

SNP^a^	Trait^b^	Chr.^c^	Position (bp)^d^	Alleles^e^	*P* value^f^	MAF^g^	FDR^h^	Effect	Markers identified using other GWAS models	Possible *Yr* gene
*S1A_11751907*	MV 1 (DS)	1A	11,751,907	**A**/G	5.92E-06	0.06	1.17E-02	8.68	FarmCPU	
*S1A_35182229*	RS 2 (DS)	1A	35,182,229	**T**/C	6.15E-06	0.18	7.58E-03	6.24	FarmCPU	
*S1A_402131971*	PL 2 (IT)	1A	402,131,971	**G**/T	2.69E-05	0.05	4.43E-02	0.85		
*S1B_306441085*	RS 2 (DS)	1B	306,441,085	**G**/A	4.04E-06	0.28	6.32E-03	5.34		*Yr26*
***S1D_244679388***	PSTV-37^a^	1D	244,679,388	**A**/G	1.58E-06	0.05	6.01E-03	1.2		
*S1D_380486701*	CH (DS)	1D	380,486,701	**G**/A	4.53E-07	0.06	1.66E-03	8.03		
*Yr17*	MV 1 (IT)	2A	5,814,790	*Non-Yr17*/***Yr17***	5.77E-31	0.49	5.70E-27	− 1.22	FarmCPU + MLM	*Yr17*
	MV 1 (DS)	2A	5,814,790	*Non-Yr17*/***Yr17***	3.42E-36	0.49	3.37E-32	− 12.32	FarmCPU + MLM	*Yr17*
*S2A_48981829*	MV 1 (IT)	2A	48,981,829	**G**/C	6.60E-06	0.35	1.30E-02	0.44	FarmCPU	
*S2A_117368947*	RS 1 (DS)	2A	117,368,947	**T**/A	1.79E-05	0.05	1.96E-02	7.66		
***S2A_177465383***	BLUE (IT)	2A	177,465,383	**C**/T	3.29E-05	0.07	4.06E-02	0.58	FarmCPU	
***S2A_310582367***	PL 3 (DS)	2A	310,582,367	**C**/A	1.39E-05	0.05	2.74E-02	9.21		
***S2A_315490348***	BLUE (DS)	2A	315,490,348	**C**/T	4.46E-06	0.14	7.90E-03	10.83		
*S2A_455472689*	PSTV-14^a^	2A	455,472,689	**T**/C	9.00E-09	0.31	1.60E-04	0.91		
***S2A_480477702***	PL 2 (IT)	2A	480,477,702	**C**/A	8.49E-06	0.12	2.79E-02	0.6		
	PL 2 (DS)	2A	480,477,702	**C**/A	5.17E-06	0.12	6.86E-03	6.45	FarmCPU	
	PL rAUDPC	2A	480,477,702	**C**/A	2.09E-07	0.12	7.58E-04	6.57		
	RS 1 (IT)	2A	480,477,702	**C**/A	2.82E-06	0.12	1.13E-02	0.52		
	BLUE (IT)	2A	480,477,702	**C**/A	2.10E-06	0.12	8.82E-03	0.5		
	BLUE (DS)	2A	480,477,702	**C**/A	8.59E-06	0.12	1.06E-02	4.42		
***S2A_485057429***	RS 1 (IT)	2A	485,057,429	**A**/G	6.60E-06	0.1	1.30E-02	0.59		
*S2A_638603325*	MV 1 (IT)	2A	638,603,325	**T**/A	1.42E-07	0.14	7.00E-04	0.71	FarmCPU	
	MV 1 (DS)	2A	638,603,325	**T**/A	2.01E-07	0.14	1.98E-03	6.74	FarmCPU	
	RS 1 (DS)	2A	638,603,325	**T**/A	4.80E-06	0.15	7.89E-03	4.49		
	BLUE (IT)	2A	638,603,325	**T**/A	2.86E-05	0.14	4.03E-02	0.41	FarmCPU	
	BLUE (DS)	2A	638,603,325	**T**/A	2.98E-05	0.14	2.94E-02	3.87		
*S2A_651459425*	MV 2 (IT)	2A	651,459,425	A/**G**	9.90E-08	0.32	4.88E-04	− 0.51	FarmCPU	
	MV 2 (DS)	2A	651,459,425	A/**G**	9.58E-07	0.32	3.15E-03	− 5.76	FarmCPU	
*S2A_658468345*	PL 1 (DS)	2A	658,468,345	**T**/G	8.63E-06	0.08	2.13E-02	4.65		
	BLUE (DS)	2A	658,468,345	**T**/G	4.81E-06	0.08	7.90E-03	5.6	FarmCPU	
*S2B_48062121*	MV 2 (IT)	2B	48,062,121	C/**T**	4.43E-09	0.21	4.37E-05	− 0.65	FarmCPU	
	MV 2 (DS)	2B	48,062,121	C/**T**	3.80E-07	0.21	3.15E-03	− 6.66	FarmCPU	
	PL 3 (IT)	2B	48,062,121	C/**T**	1.01E-05	0.21	2.49E-02	− 0.55	FarmCPU	
	BLUE (IT)	2B	48,062,121	C/**T**	1.16E-05	0.21	1.90E-02	− 0.37		
*S2B_607798364*	PL 1 (IT)	2B	607,798,364	**G**/A	2.31E-06	0.12	1.14E-02	0.65		*Yr53*
	PL 2 (DS)	2B	607,798,364	**G/**A	2.05E-06	0.12	3.37E-03	6.75	FarmCPU	*Yr53*
	PL rAUDPC	2B	607,798,364	**G**/A	8.42E-07	0.12	2.08E-03	6.37	FarmCPU	*Yr53*
*S2B_625334668*	RS 1 (IT)	2B	625,334,668	**A**/G	4.46E-09	0.07	4.30E-05	0.84	FarmCPU	
	BLUE (IT)	2B	625,334,668	**A**/G	4.39E-06	0.08	8.82E-03	0.58		
*S2D_122329962*	MV 1 (IT)	2D	122,329,962	**C/**T	3.72E-06	0.13	9.18E-03	0.67	FarmCPU	
	MV 1 (DS)	2D	122,329,962	**C**/T	3.58E-06	0.13	8.83E-03	6.42	FarmCPU	
***S2D_445703860***	PSTV-14^a^	2D	445,703,860	**T**/C	2.22E-06	0.05	9.87E-03	1.23		
***S3A_128420383***	PSTV-37^a^	3A	128,420,383	**G**/A	2.96E-06	0.19	8.76E-03	0.7		
	CH (DS)	3A	128,420,383	**G**/A	1.59E-05	0.12	1.74E-02	4.78		
	RS 1 (DS)	3A	128,420,383	**G**/A	2.19E-06	0.12	4.32E-03	5.2	FarmCPU	
***S3A_204766888***	RS 2 (DS)	3A	204,766,888	**G**/A	2.70E-07	0.1	8.89E-04	9.29		
***S3A_261175449***	PL 3 (DS)	3A	261,175,449	**G**/T	9.59E-08	0.09	9.45E-04	8.97	FarmCPU	
	RS 1 (DS)	3A	261,175,449	**G**/T	1.22E-08	0.09	1.20E-04	6.71	FarmCPU	
	RS 2 (DS)	3A	261,175,449	**G**/T	4.58E-09	0.09	2.26E-05	12.03	FarmCPU	
	RS rAUDPC	3A	261,175,449	**G**/T	1.17E-08	0.09	1.15E-04	8.88	FarmCPU	
	BLUE (DS)	3A	261,175,449	**G**/T	4.11E-08	0.09	1.35E-04	6.71	FarmCPU	
***S3A_306830730***	PL 2 (IT)	3A	306,830,730	**G**/A	2.97E-07	0.11	2.93E-03	0.75	FarmCPU	
	BLUE (IT)	3A	306,830,730	**G**/A	4.47E-06	0.11	8.82E-03	0.5	FarmCPU	
***S3A_310982989***	PL 2 (DS)	3A	310,982,989	**C**/G	4.14E-05	0.05	3.71E-02	8.32		
***S3A_434787689***	CH (DS)	3A	434,787,689	**C**/T	5.04E-07	0.05	1.66E-03	8.27		
***S3A_470848383***	RS 1 (DS)	3A	470,848,383	**T**/G	8.95E-07	0.05	4.32E-03	8.21	FarmCPU	
*S3A_499526052*	RS 2 (DS)	3A	499,526,052	**G**/A	9.18E-11	0.16	9.05E-07	9.99		
*S3A_507928475*	PL 1 (IT)	3A	507,928,475	G/**A**	6.09E-06	0.15	2.00E-02	−0.5		
*S3A_509438360*	PL 2 (DS)	3A	509,438,360	**G**/T	1.95E-05	0.37	2.14E-02	3.94		
*S3A_577488099*	PL 1 (DS)	3A	577,488,099	**A**/T	6.82E-06	0.05	2.13E-02	6.36	FarmCPU	
***S3A_617671922***	PL 1 (DS)	3A	617,671,922	**T**/C	2.48E-05	0.24	4.66E-02	2.69		
*S3B_35280404*	RS rAUDPC	3B	35,280,404	**A**/G	1.81E-06	0.07	8.90E-03	7.57	FarmCPU	
*S3B_35986723*	CH (DS)	3B	35,986,723	**G**/A	3.28E-06	0.24	4.63E-03	4.05		
*S3B_220508469*	CH (DS)	3B	220,508,469	**G**/T	2.45E-06	0.08	4.63E-03	6.62		
*S3B_646995777*	PL 2 (DS)	3B	646,995,777	G/**T**	1.84E-06	0.06	3.37E-03	−9.52	FarmCPU	
*S3B_800750041*	MV 1 (IT)	3B	800,750,041	**A**/G	1.35E-06	0.19	4.45E-03	0.59		
	MV 1 (DS)	3B	800,750,041	**A**/G	8.74E-07	0.19	4.31E-03	5.94		
*S3D_506908634*	PL 1 (DS)	3D	506,908,634	**G**/T	2.84E-05	0.06	4.66E-02	4.94	FarmCPU	
***S4A_271844588***	RS 1 (IT)	4A	271,844,588	**C**/A	1.69E-05	0.06	2.34E-02	0.69		
***S4A_352174561***	RS 1 (IT)	4A	352,174,561	C/**T**	4.59E-06	0.07	1.13E-02	−2.38		
	RS 1 (DS)	4A	352,174,561	C/**T**	1.54E-06	0.07	4.32E-03	−24.1		
***S4B_68391161***	PL 2 (IT)	4B	68,391,161	**T**/C	2.79E-06	0.48	1.37E-02	0.44	FarmCPU	
	PL 2 (DS)	4B	68,391,161	**T**/C	1.25E-07	0.48	6.16E-04	5.05	FarmCPU	
	PL rAUDPC	4B	68,391,161	**T**/C	2.33E-06	0.48	3.82E-03	3.97		
***S4B_436289930***	RS 1 (IT)	4B	436,289,930	**A**/G	4.27E-06	0.05	1.13E-02	0.78	FarmCPU	
*S4B_526319279*	PSTV-37^a^	4B	526,319,279	**G**/C	1.69E-06	0.06	6.01E-03	1.29		*Yr62*
*S4B_560661391*	RS 2 (DS)	4B	560,661,391	**G**/A	2.34E-05	0.08	2.31E-02	8.62		*Yr62*, *Yr68*
*S4B_571886653*	PSTV-37^a^	4B	571,886,653	**T**/C	1.06E-07	0.15	9.38E-04	0.87		*Yr62*, *Yr68*
	PL 2 (IT)	4B	571,886,653	**T**/C	1.88E-05	0.11	3.71E-02	0.62		*Yr62*, *Yr68*
	PL 2 (DS)	4B	571,886,653	**T**/C	2.49E-09	0.11	2.45E-05	9.89	FarmCPU	*Yr62*, *Yr68*
	PL 3 (IT)	4B	571,886,653	**T**/C	9.84E-06	0.11	2.49E-02	0.73		*Yr62*, *Yr68*
	PL 3 (DS)	4B	571,886,653	**T**/C	6.66E-07	0.11	3.28E-03	8.67	MLM	*Yr62*, *Yr68*
	PL rAUDPC	4B	571,886,653	**T**/C	2.78E-10	0.11	2.74E-06	9.38	FarmCPU + MLM	*Yr62*, *Yr68*
	BLUE (IT)	4B	571,886,653	**T**/C	3.73E-08	0.11	3.68E-04	0.65		*Yr62*, *Yr68*
*S4D_196312261*	RS 2 (DS)	4D	196,312,261	**A**/G	6.67E-07	0.07	1.64E-03	10.8	FarmCPU	
***S4D_297199807***	PSTV-37^a^	4D	297,199,807	**C**/A	1.18E-06	0.08	6.01E-03	1.04		
***S5A_167153999***	CH (DS)	5A	167,153,999	C/**T**	1.13E-06	0.13	2.79E-03	− 5.41		
***S5A_326665354***	MV 2 (IT)	5A	326,665,354	**A**/G	7.35E-06	0.05	1.81E-02	0.94	FarmCPU	
*S5A_528446200*	MV 2 (DS)	5A	528,446,200	**G**/A	2.78E-05	0.06	4.56E-02	9.39		
*S5B_85433288*	MV 1 (IT)	5B	85,433,288	**C**/G	2.23E-05	0.28	3.67E-02	0.44		
*S5B_516675208*	PL 2 (DS)	5B	516,675,208	**G**/A	5.57E-06	0.09	6.86E-03	6.98	FarmCPU	
***S5D_90288936***	MV 2 (DS)	5D	90,288,936	**G**/T	3.74E-05	0.05	5.26E-02	9.72	FarmCPU	
***S5D_93017359***	MV 1 (IT)	5D	93,017,359	**T**/C	6.42E-09	0.06	6.33E-05	1.16	FarmCPU	
***S5D_282820006***	RS 2 (DS)	5D	282,820,006	T/**G**	2.17E-06	0.32	4.28E-03	− 5.31		
	BLUE (DS)	5D	282,820,006	T/**G**	1.52E-06	0.33	3.76E-03	− 3.43	FarmCPU	
*S6A_110832428*	MV 1 (DS)	6A	110,832,428	A/**C**	3.37E-06	0.34	8.83E-03	− 4.03		
***S6A_257790349***	RS 1 (DS)	6A	257,790,349	**C**/G	2.08E-06	0.16	4.32E-03	4.27	FarmCPU	
	RS 2 (DS)	6A	257,790,349	**C**/G	9.58E-06	0.17	1.05E-02	7.16	FarmCPU	
	BLUE (DS)	6A	257,790,349	**C**/G	3.74E-08	0.17	1.35E-04	5.14		
***S6A_435521395***	PL 3 (IT)	6A	435,521,395	**G**/A	3.78E-06	0.48	2.49E-02	0.45		
*S6A_502636146*	RS 1 (IT)	6A	502,636,146	**G**/A	1.90E-05	0.15	2.34E-02	0.46		
*S6A_518030624*	RS 1 (DS)	6A	518,030,624	**C**/T	1.01E-05	0.12	1.42E-02	4.75		
***S7A_43649084***	PSTV-37^a^	7A	43,649,084	**A**/T	1.47E-12	0.07	2.61E-08	1.72		
*S7A_260372780*	RS 1 (IT)	7A	260,372,780	**T**/A	1.41E-05	0.05	2.32E-02	0.83	FarmCPU	
***S7A_329846886***	PL 1 (DS)	7A	329,846,886	G/**A**	3.85E-07	0.3	3.79E-03	− 3.17	FarmCPU	
	PL 2 (DS)	7A	329,846,886	G/**A**	1.34E-06	0.3	3.31E-03	− 4.84		
	PL 3 (IT)	7A	329,846,886	G/**A**	6.74E-06	0.3	2.49E-02	− 0.49		
	PL rAUDPC	7A	329,846,886	G/**A**	1.57E-06	0.3	3.09E-03	− 4.36		
	BLUE (IT)	7A	329,846,886	G/**A**	3.96E-06	0.3	8.82E-03	− 0.35		
*S7A_441278018*	PL 3 (DS)	7A	441,278,018	**C**/T	3.62E-06	0.06	8.92E-03	9.18		
	RS 1 (DS)	7A	441,278,018	**C**/T	2.91E-05	0.06	2.86E-02	6.28	FarmCPU	
***S7A_464464915***	PL 1 (DS)	7A	464,464,915	**C**/T	4.37E-06	0.08	2.13E-02	4.83	FarmCPU	
	PL 2 (IT)	7A	464,464,915	**C**/T	1.22E-05	0.08	3.02E-02	0.66		
	PL 3 (DS)	7A	464,464,915	**C**/T	1.77E-06	0.08	5.83E-03	8.14		
*S7A_501355562*	PL 1 (IT)	7A	501,355,562	**A**/G	1.86E-09	0.35	1.83E-05	0.58		
	BLUE (DS)	7A	501,355,562	**A**/G	1.75E-05	0.35	1.91E-02	2.93		
*S7A_548774801*	MV 2 (DS)	7A	548,774,801	G/**A**	6.92E-07	0.39	3.15E-03	− 5.39	FarmCPU	
*S7A_556685233*	RS 1 (DS)	7A	556,685,233	**T**/A	1.37E-05	0.09	1.69E-02	5.29		
*S7B_49808365*	MV 2 (IT)	7B	49,808,365	A/**G**	7.95E-07	0.2	2.61E-03	− 0.57	FarmCPU	
	MV 2 (DS)	7B	49,808,365	A/**G**	6.33E-06	0.2	1.38E-02	− 6.43	FarmCPU	
*S7B_55209301*	RS 2 (DS)	7B	55,209,301	A/**G**	4.49E-06	0.18	6.32E-03	− 6.25		
*S7B_461525341*	PSTV-14^a^	7B	461,525,341	T/**C**	3.76E-07	0.15	3.34E-03	− 0.92	FarmCPU	
*S7D_13114223*	PL 3 (DS)	7D	13,114,223	**G**/A	1.80E-05	0.29	2.96E-02	4.26		
*S7D_119510231*	MV 1 (DS)	7D	119,510,231	G/**A**	3.20E-05	0.15	4.51E-02	− 4.91		
***S7D_213389169***	PSTV-14^a^	7D	213,389,169	**C**/A	1.54E-06	0.06	9.08E-03	1.18	FarmCPU	
***S7D_279512503***	CH (DS)	7D	279,512,503	**A**/G	2.84E-06	0.05	4.63E-03	8		
***S7D_449212105***	PL 2 (DS)	7D	449,212,105	**G**/A	2.79E-07	0.11	9.16E-04	8.03	FarmCPU	
	PL rAUDPC	7D	449,212,105	**G**/A	2.31E-07	0.11	7.58E-04	6.28	FarmCPU	
*S7D_518590833*	CH (DS)	7D	518,590,833	**G**/A	4.15E-07	0.07	1.66E-03	7.61		
	PL 2 (DS)	7D	518,590,833	**G**/A	3.81E-05	0.07	3.71E-02	7.72	FarmCPU	
	BLUE (DS)	7D	518,590,833	**G**/A	6.47E-06	0.07	9.11E-03	6.14	FarmCPU	

^a*^SNPs associated with both seedling and adult plant stage; the SNPs in bold are potentially novel SNPs based on comparison with previously known *Yr* genes and QTLs

^b^Trait code (a = infection type at the adult plant stage; IT = infection type; DS = disease severity (%); CH = Chickasha, OK; MV 1 = first disease rating at Mount Vernon, WA; MV 2 = second disease rating at Mount Vernon, WA; PL 1 = first disease rating at Pullman, WA; PL 2 = second disease rating at Pullman, WA; PL 3 = third disease rating at Pullman; PL rAUDPC = relative area under disease progress curve calculated based on Pullman DS data; RS 1 = first disease rating at Rossville, KS; RS 2 = second disease rating at Rossville, KS; RS rAUDPC = relative area under disease progress curve calculated based on Rossville DS data; BLUE = multi-environment best linear unbiased estimates)

^c^Chromosome

^d^Physical position of SNP sequence based on Wheat Chinese Spring IWGSC RefSeq v2.1 (Zhu et al. 2021). Physical positions of characterized *Yr* genes/QTL were based onTong et al. (2024)

^e^SNP major allele/minor allele, the allele in bold is associated with stripe rust resistance

^f^*P* value of the significant SNP

^g^Minor allele frequency of the significant SNP

^h^False discovery rate of the significant SNP

### Marker-trait associations

**Fig. 4:**
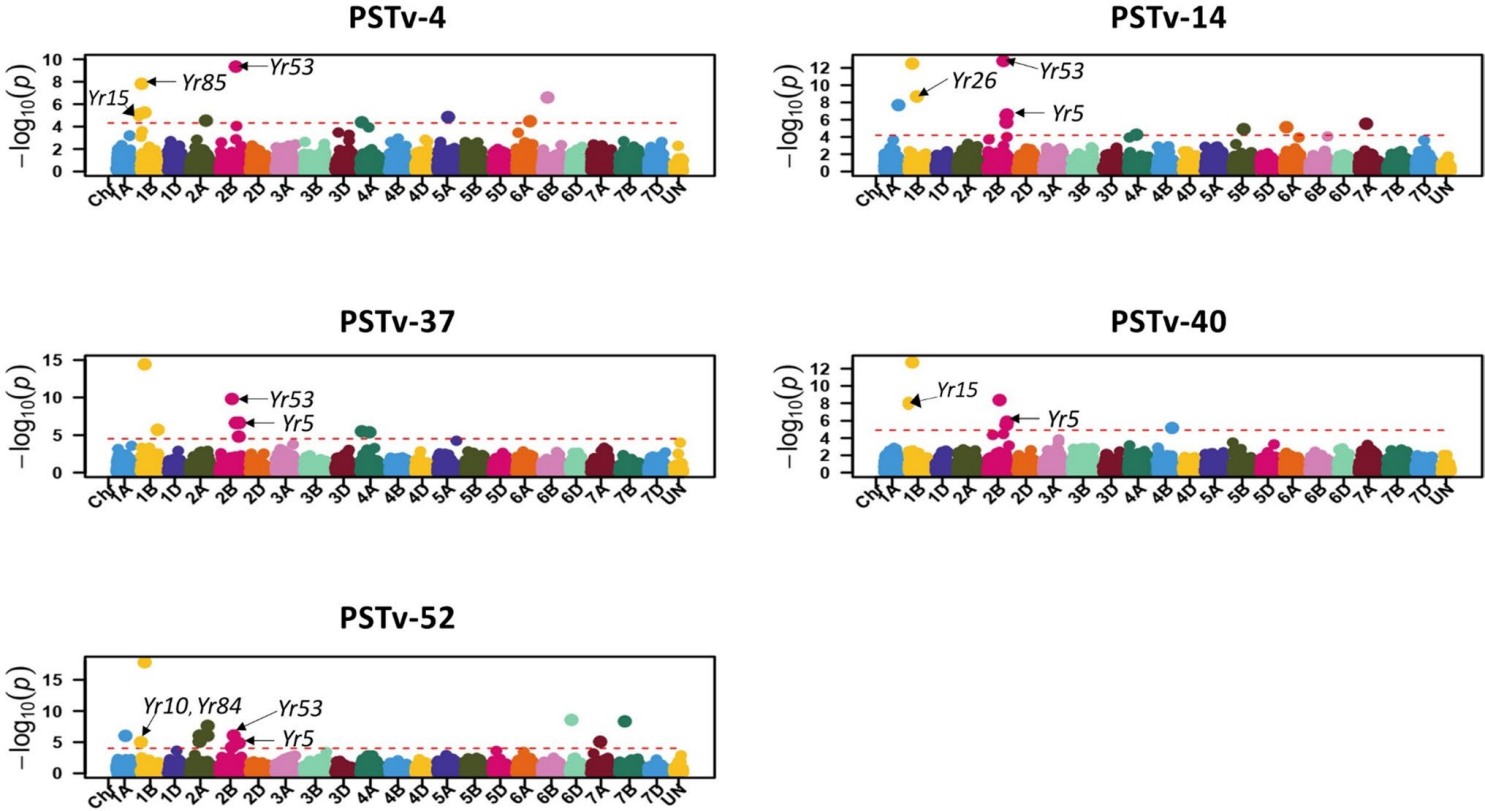
Manhattan plots showing significant SNPs associated with stripe rust response at the seedling stage to five *Puccinia striiformis* f. sp*. tritici* races using the BLINK model. The horizontal red line indicates significance levels at a false discovery rate ≤ 0.05 (colour figure online)

A total of 110 significant SNPs associated with stripe rust response and corresponding to 109 unique QTL were identified across all 21 wheat chromosomes using the BLINK model (Tables 3 and 4). Of these significant SNPs, 59 were located on the A genome, 33 on the B genome, and 17 on the D genome. Chromosome 2A harbored the highest number of significant SNPs (*n* = 16), followed by chromosome 3A (*n* = 12), whereas chromosomes 3D, 6B, and 6D each contained a single significant SNP. Details on stripe rust-resistant alleles for each significant SNP and their effects are outlined in Tables 3 and 4. Markers linked to five *Yr* genes/QTL, *Yr15*, *Yr17*, *Yr18*, *Yr29*, and *QYr.tamu-2B*, were included in the GWAS. Based on functional DNA markers, *Yr5*, *Yr36*, *Yr40*, and *Yr46* were absent or present at low frequencies (< 5%) in this HWW panel (Fig. 3) and thus were excluded from the GWAS. Of the 110 significant SNPs, 31 were associated with seedling responses with the highest number of significant SNPs associated with response to race PSTv-52 and the fewest number of significant SNPs associated with response to race PSTv-40 (Table 3 and Fig. 4)*.* Marker linked to *Yr15* was associated with stripe rust response to races PSTv-4 and PSTv-40. Among the 31 identified SNPs at the seedling stage using the BLINK model, 21 were also detected using other models (FarmCPU, MLM, or both), further validating these MTAs identified by the BLINK model.

**Fig. 5:**
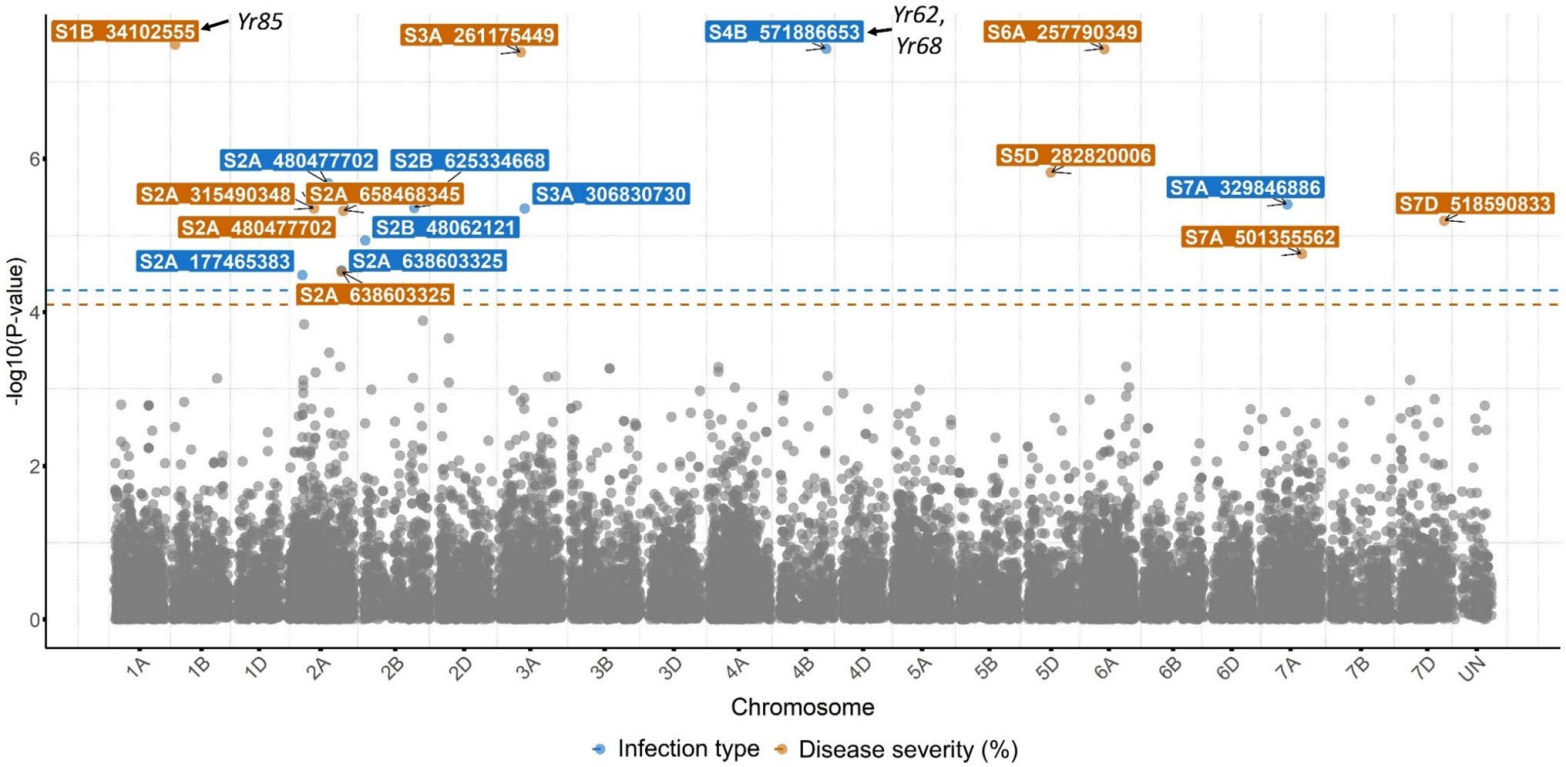
Manhattan plots showing significant SNPs associated with multi-environment best linear unbiased estimates for stripe rust infection type and disease severity at the adult plant stage using the BLINK model. The horizontal orange and blue lines indicate significance levels at a false discovery rate ≤ 0.05 (colour figure online)

At the adult plant stage, 79 SNPs were distributed across all wheat chromosomes, except 6B and 6D, which were significantly associated with stripe rust response across field environments (Table 4). There were 8, 32, 44, and 25 SNPs significantly associated with stripe rust response at Chickasha, Rossville, Pullman, and Mount Vernon, respectively. Multi-environment BLUE revealed eight SNPs associated with IT and 10 SNPs associated with DS (Table 4, Fig. 5). Of those 18 SNPs, 16 were associated with at least one other trait of stripe rust response. The marker linked to *Yr17* was among the significant associations for each MV 1 (IT) and MV 1 (DS). GWAS identified four and six significant SNPs associated with IT at the adult plant stage to races PSTv-14 and PSTv-37, respectively. No significant associations were found for IT at the adult plant stage to race PSTv-40. The significant SNP *S1B_7915711*, associated with seedling response, and the SNP *S1B_7245353*, associated with adult plant stage response at Chickasha, are both located on the short arm of chromosome 1B and are in LD (*r*^*2*^ ≥ 0.1), thus representing the same QTL (Table 3). FarmCPU and/or MLM further validated 40 out of the identified 79 SNPs associated with the adult plant stage response to stripe rust using the BLINK model.

Among the 110 SNPs identified in this study, 31 were associated with stripe rust response in more than one test/environment (Table 5). Of these, 28 (90%) were also validated by either FarmCPU, MLM, or both models. Three significant SNPs, *S1B_34102555*, *S1B_141655194*, and *S2B_724829085*, were associated with stripe rust response at both the seedling and adult plant stages. Five SNPs, *S1B_141655194*, *S2B_458606839*, S*2B_598744752*, *S2B_706506028,* and *S2B_724829085*, were associated with seedling IT against more than three *Pst* races. Marker *S1B_141655194* was associated with seedling IT against all five *Pst* races and exhibited the lowest *P*-values. Furthermore, seven significant SNPs identified at the adult plant stage, *S2A_480477702*, *S2A_638603325*, *S2B_48062121*, *S3A_261175449*, *S4B_571886653*, *S7A_329846886*, and *S7D_518590833*, demonstrated high stability as they were associated with stripe rust responses in at least three field environments. Of the 459 genotypes, 32 carried as many as 79–85 favorable alleles of the 110 SNPs significantly associated with stripe rust response in this study (Supplementary Table S8).

**Table 5:** List of 31 SNP markers associated with stripe rust response in multiple tests/environments based on the BLINK GWAS model

SNP^a^	Chr.^b^	Position (bp)^c^	Alleles^d^	MAF^e^	Trait^f^	Other model associations
*S1B_34102555*	1B	34,102,555	A/**G**	0.06	PSTv-4^s^, BLUE (DS)	FarmCPU + MLM
*S1B_141655194*	1B	141,655,194	T/**G**	0.05	PSTv-4^s^, PSTv-14^s^, PSTv-37^s^, PSTv-40^s^, PSTv-52^s^, MV 1 (DS)	FarmCPU + MLM
***S2A_480477702***	2A	480,477,702	**C**/A	0.12	PL 2 (IT), PL 2 (DS), PL rAUDPC, RS 1 (IT), BLUE (IT), BLUE (DS)	FarmCPU
*S2A_638603325*	2A	638,603,325	**T**/A	0.14	MV 1 (IT), MV 1 (DS), RS 1 (DS), BLUE (IT), BLUE (DS)	FarmCPU
*S2A_651459425*	2A	651,459,425	A/**G**	0.32	MV 2 (IT), MV 2 (DS)	FarmCPU
*S2A_658468345*	2A	658,468,345	**T**/G	0.08	PL 1 (DS), BLUE (DS)	FarmCPU
*S2B_48062121*	2B	48,062,121	C/**T**	0.21	MV 2 (IT), MV 2 (DS), PL 3 (IT), BLUE (IT)	FarmCPU
*S2B_458606839*	2B	458,606,839	A/**G**	0.08	PSTv-37^s^, PSTv-40^s^, PSTv-52^s^	FarmCPU + MLM
*S2B_598744752*	2B	598,744,752	T/**C**	0.05	PSTv-4^s^, PSTv-14^s^, PSTv-37^s^, PSTv-52^s^	FarmCPU + MLM
*S2B_607798364*	2B	607,798,364	**G**/A	0.12	PL 1 (IT), PL 2 (DS), PL rAUDPC	FarmCPU
*S2B_625334668*	2B	625,334,668	**A**/G	0.07	RS 1 (IT), BLUE (IT)	FarmCPU
*S2B_706506028*	2B	706,506,028	T/**C**	0.09	PSTv-14^s^, PSTv-37^s^, PSTv-40^s^, PSTv-52^s^	MLM
*S2B_724829085*	2B	724,829,085	T/**A**	0.10	PSTv-14^s^, PSTv-37^s^, PSTv-40^s^, MV 2 (DS)	FarmCPU + MLM
*S2D_122329962*	2D	122,329,962	**C/**T	0.13	MV 1 (IT), MV 1 (DS)	FarmCPU
***S3A_128420383***	3A	128,420,383	**G**/A	0.19	PSTV-37^a^, CH (DS), RS 1 (DS)	FarmCPU
***S3A_261175449***	3A	261,175,449	**G**/T	0.09	PL 3 (DS), RS 1 (DS), RS 2 (DS), RS rAUDPC, BLUE (DS)	FarmCPU
***S3A_306830730***	3A	306,830,730	**G**/A	0.11	PL 2 (IT), BLUE (IT)	FarmCPU
*S3B_800750041*	3B	800,750,041	**A**/G	0.19	MV 1 (IT), MV 1 (DS)	
***S4A_61065066***	4A	61,065,066	**G**/T	0.05	PSTv-4^s^, PSTv-37^s^	FarmCPU
***S4A_352174561***	4A	352,174,561	C/**T**	0.07	RS 1 (IT), RS 1 (DS)	
***S4B_68391161***	4B	68,391,161	**T**/C	0.48	PL 2 (IT), PL 2 (DS), PL rAUDPC	FarmCPU
*S4B_571886653*	4B	571,886,653	**T**/C	0.11	PSTv-40^s^, PSTV-37^a^, PL 2 (IT), PL 2 (DS), PL 3 (IT), PL 3 (DS), PL rAUDPC, BLUE (IT)	FarmCPU + MLM
***S5D_282820006***	5D	282,820,006	T/**G**	0.32	RS 2 (DS), BLUE (DS)	FarmCPU
***S6A_257790349***	6A	257,790,349	**C**/G	0.16	RS 1 (DS), RS 2 (DS), BLUE (DS)	FarmCPU
***S7A_329846886***	7A	329,846,886	G/**A**	0.30	PL 1 (DS), PL 2 (DS), PL 3 (IT), PL rAUDPC, BLUE (IT)	FarmCPU
*S7A_441278018*	7A	441,278,018	**C**/T	0.06	PL 3 (DS), RS 1 (DS)	FarmCPU
***S7A_464464915***	7A	464,464,915	**C**/T	0.08	PL 1 (DS), PL 2 (IT), PL 3 (DS)	FarmCPU
*S7A_501355562*	7A	501,355,562	**A**/G	0.35	PL 1 (IT), BLUE (DS)	
*S7B_49808365*	7B	49,808,365	A/**G**	0.20	MV 2 (IT), MV 2 (DS)	FarmCPU
***S7D_449212105***	7D	449,212,105	**G**/A	0.11	PL 2 (DS), PL rAUDPC	FarmCPU
*S7D_518590833*	7D	518,590,833	**G**/A	0.07	CH (DS), PL 2 (DS), BLUE (DS)	FarmCPU

^a^The SNPs in bold are potentially novel SNPs based on comparison with previously known *Yr* genes and QTL

^b^Chromosome

^c^Physical position of SNP sequence based on Wheat Chinese Spring IWGSC RefSeq v2.1 (Zhu et al. 2021)

^d^SNP major allele/minor allele, the allele in bold is associated with stripe rust resistance

^e^Minor allele frequency of the significant SNP

^f^Trait code (s = infection type at seedling stage; a = infection type at the adult plant stage; IT = infection type; DS = disease severity (%); CH = Chickasha, OK; MV 1 = first disease rating at Mount Vernon, WA; MV 2 = second disease rating at Mount Vernon, WA; PL 1 = first disease rating at Pullman, WA; PL 2 = second disease rating at Pullman, WA; PL 3 = third disease rating at Pullman; PL rAUDPC = relative area under disease progress curve calculated based on Pullman DS data; RS 1 = first disease rating at Rossville, KS; RS 2 = second disease rating at Rossville, KS; RS rAUDPC = relative area under disease progress curve calculated based on Rossville DS data; BLUE = multi-environment best linear unbiased estimates

## Discussion

The virulent races emerging post-2000 have rendered the majority of ASR genes and a few of the APR genes deployed in wheat varieties ineffective (Hovmøller et al. 2011; Sørensen et al. 2014; Wan and Chen 2014; Mu et al. 2020). In this study, the HWW panel exhibited high susceptibility at the seedling stage and moderate to high resistance at the adult plant stage, indicating the presence of APR in the panel. Similar observations were reported in various US wheat panels (Liu et al. 2018, 2020; Mu et al. 2020; Aoun et al. 2021b). Furthermore, the differential virulence levels of *Pst* races to the NRPN and SRPN genotypes at the adult plant stage suggest the presence of race-specific APR genes in these genotypes. In comparison with stripe rust responses at Chickasha, OK, and Rossville, KS, lower levels of resistance were observed in the HWW panel in Mount Vernon and Pullman, WA, where the climate is cooler and more humid, and where *Pst* virulence diversity is higher. Kansas and Oklahoma have typically warmer climates, which may have contributed to the activation of HTAP resistance genes witnessed herein. Another reason could be genotype × environment interactions that may have influenced the expression of APR genes. For instance, the pleiotropic APR gene *Lr34*/*Yr18* present in 17% of genotypes in the current HWW panel, has been shown to exhibit variable levels of gene expression affected by genetic background, infection levels, and environmental factors (Risk et al. 2012).

Sixteen genotypes demonstrated resistance to the five tested *Pst* races at the seedling stage and exhibited stable resistance at the adult plant stage in field environments. These genotypes are valuable ASR sources for breeding programs given the lack of ASR in contemporary US HWW cultivars. Although cultivars with multiple APR genes can have sufficient protection at the adult plant stage, early and severe stripe rust epidemics in the southern and central US Great Plains in 2024 caused substantial yield losses if planted cultivars were not treated with fungicides. Diagnostic molecular markers revealed that most of these resistant genotypes carried the broad-spectrum ASR genes *Yr5* and/or *Yr15*. Originally identified in *T. aestivum* subsp. *spelta* cv. Album, *Yr5* was not utilized in commercial wheat cultivars due to its linkage drag associated with yield until recent efforts to reincorporate it (Wang and Chen 2017). Similarly, *Yr15* was initially identified in wild emmer wheat, *T. turgidum* var. *dicoccoides*, accession G25 (Gerechter-Amitai et al. 1989) and has yet to be deployed in commercial cultivars in the Great Plains. Wheat breeding programs in the Great Plains have recently begun to incorporate *Yr5* and *Yr15* in tandem, explaining why only 4% and 6% of HWW genotypes in this study carry *Yr5* and *Yr15*, respectively. Mu et al. (2020) reported similar findings in US winter wheat cultivars and breeding lines, where *Yr5* was present at low frequencies, and *Yr15* was absent. The deployment of *Yr5* and *Yr15* together in wheat cultivars is recommended to reduce the risk of emergence of virulent races to these valuable resistance genes. Although *Yr5* and *Yr15* identified in this HWW panel are highly stable ASR genes in the USA (Wan and Chen 2014; Wang et al. 2022), relying solely on these two ASR genes would not be a wise or durable approach, as the pathogen could quickly evolve, and virulent races might emerge. For instance, *Pst* races virulent to *Yr5* have been reported in India, Australia, China, and Turkey (Tekin et al. 2021). Among the 16 genotypes with broad-spectrum ASR, the breeding lines CO19D304R and TX18DH266 did not carry *Yr5* and *Yr15*. These two lines were also included in the 2023 SRPN, and based on molecular markers linked to known *Yr* genes, CO19D304R was heterozygous for *Yr78*, whereas TX18DH266 carried *Yr29*/*Lr46*, *Yr78*, and *Q.Yr.tamu-2B*. However, the APR genes *Yr29*/*Lr46* and *Yr78* provide partial APR (Singh et al. 1998; William et al. 2003; Dong et al. 2017), and *Q.Yr.tamu-2B* provides race-specific ASR (Basnet et al. 2014). Therefore, the high resistance levels in these two genotypes against multiple *Pst* races and across field environments might be due to the presence of other unknown *Yr* genes that can be used to diversify the narrow genetic basis of ASR in HWW.

This GWAS identified the SNP marker *S2B_706506028* to be associated with response against all tested *Pst* races except PSTv-4. This marker was located within the genomic region of *Yr5* (~ 700,891,0000 bp) and was in LD with the diagnostic marker for *Yr5* (*r*^*2*^ = 0.2); thus, *S2B_706506028* could be associated with *Yr5* or *YrSP*, which is allelic to *Yr5* (Marchal et al. 2018; Wan and Chen 2014; Wan et al. 2016)*. S2B_706506028* was present at higher frequency of 9% compared to 4% for the diagnostic marker for *Yr5*, which got filtered out from the GWAS because of its low MAF. The high frequencies of *Yr17* and *Yr29* indicate that these genes have been extensively used in breeding programs in the Great Plains. *Yr17* located on the 2NS/2AS translocation (Helguera et al. 2003) is ineffective against most current *Pst* races. Although *Yr17* was not detected in our GWAS at the seedling stage because it is ineffective against all tested races except PSTv-40 (Wan and Chen 2014; Wan et al. 2016), it was identified in the field environment at Mount Vernon. The field resistance should be attributed to an HTAP resistance gene *YrM1225* closely linked to *Yr17* (Li et al. 2023).

Based on the physical positions of molecular markers on the reference genome IWGSC_RefSeq v2.1 (Zhu et al. 2021), we determined the relationship between previously reported *Yr* genes/QTL and significant SNPs associated with stripe rust response in this study. Significant SNPs were deemed novel if they were not located within the genomic regions of known *Yr* genes/QTL (≥ 15 Mb). We compared the significant SNPs identified in this study to 86 previously reported *Yr* genes, 77 temporarily named *Yr* genes, and over 325 QTL associated with stripe rust resistance reviewed by Wang and Chen (2017), McIntosh et al. (2020), and Zhu et al. (2023). Furthermore, over 600 QTL, reported in 28 previous association mapping, QTL mapping, meta-QTL analysis studies conducted between 2017 and 2024 (Liu et al. 2018; Ledesma-Ramírez et al. 2019; Miedaner et al. 2019; Genievskaya et al. 2020; Habib et al. 2020; Jia et al. 2020; Juliana et al. 2020; Kumar et al. 2020; Liu et al. 2020; Mu et al. 2020; Muleta et al. 2020; Alemu et al. 2021; Aoun et al. 2021c; Tehseen et al. 2021; Zhang et al. 2021; Tomar et al. 2021; Wang et al. 2021; Yao et al. 2021; Franco et al. 2022; Jambuthenne et al. 2022; Shahinnia et al. 2022; El Messoadi et al. 2023; Mustahsan et al. 2023; Kumar et al. 2023; Yang et al. 2023; El Messoadi et al. 2024; Qiao et al. 2024) were utilized for our comparative mapping.

Ten significant SNPs from this study were identified within genomic regions of characterized *Yr* genes including *Yr5/YrSP*, *Yr10*, *Yr15*, *Yr26*, *Yr53*, *Yr62*, *Yr68*, *Yr84*, and *Yr85* (Supplementary Table S9). Two significant SNPs, *S1B_7915711* and *S1B_7245353*, associated with stripe rust response at the seedling stage, were found in proximity to the ASR genes *Yr10* (~ 5.5 Mb) and *Yr84* (~ 10 Mb) (Liu et al. 2014; Klymiuk et al. 2022). Another significant SNP, *S1B_34102555*, associated with stripe rust response at both the seedling and adult plant stages, was mapped within the genomic region of *Yr85*, which was first identified in the winter club wheat cultivar “Tres” and previously known as *YrTr1* (Feng et al. 2023). *Yr85* confers race-specific ASR against PSTv-4, and SNP *S1B_34102555* was also found to be associated with response against only PSTv-4 at the seedling stage; thus, *S1B_34102555* is likely associated with *Yr85.* The significant SNPs *S1B_308703553* associated with seedling response stage and *S1B_306441085* associated with adult plant stage response were mapped at the proximity of *Yr26* (= *Yr24*), which originated from the Chinese landrace *T. turgidum* Gamma 80-1 (Ma et al. 2001). Although *S1B_308703553* and *S1B_306441085* are physically close, LD analysis confirmed that they are two distinct loci. The significant markers *S2B_598744752* and *S2B_607798364* overlap the genomic region of *Yr53*. *Yr53*, derived from durum wheat, was mapped on chromosome 2B (605.8 Mb) and confers resistance to all US *Pst* races at the seedling stage (Xu et al. 2013; Jambuthenne et al. 2022). Similarly, *S2B_598744752* was also found to be associated with response to multiple *Pst* races. *S2B_607798364* was identified at the adult plant stage in multiple environments, and it is also likely to be associated with *Yr53*. The significant markers,* S4B_526319279*, *S4B_560661391*, and *S4B_571886653*, associated with stripe rust response at the adult plant stage, were found proximal to *Yr62* (Lu et al. 2014) and *Yr68* (McIntosh et al. 2016; Wang and Chen 2017). *Yr62* confers HTAP resistance and was identified in the Portuguese spring wheat variety PI 192252, and *Yr68* confers APR. These results indicate the successful incorporation of *Yr53*, *Yr62*, and *Yr68* into some of the HWW breeding lines.

Comparative mapping with previous GWAS and QTL mapping studies for stripe rust resistance revealed that 70 SNPs were co-localized within genomic regions of previously identified *Yr* genes/QTL, while 40 were located in regions not previously known to harbor stripe rust resistance genes/loci and thus were considered novel (Supplementary Table S9). Of the 31 SNPs associated with seedling stripe rust response, seven are likely associated with novel stripe rust resistance loci, which are *S2A_362922651*, *S2A_367020239*, *S4A_61065066*, *S4A_337252413*, *S5A_392096163*, *S7A_339701189,* and *S7B_206209604*. Of the 79 SNPs associated with stripe rust resistance at the adult plant stage, 33 SNPs are likely associated with novel stripe rust resistance loci. The discovery of these 40 novel stripe rust resistance loci should enhance breeding for stripe rust resistance in wheat and diversify sources of resistance. Additionally, of the 31 SNPs that were associated with multiple tests/environments, 12 SNPs were likely associated with novel stripe rust resistance loci. These 31 SNPs can be converted to competitive allele-specific PCR (KASP) markers or thermal asymmetric reverse PCR (STARP) markers for potential use in marker-assisted breeding.

In conclusion, this study is the first to comprehensively investigate stripe rust resistance loci in a large panel of contemporary US HWW. We revealed a high prevalence of APR and limited ASR sources in this panel. Sixteen genotypes with a broad spectrum of resistance were identified, which can serve as valuable ASR sources in wheat breeding programs. Based on functional DNA markers for some known *Yr* genes/QTL, *Yr5*, *Yr15*, *Yr17*, *Yr18*, *Yr29*, and *QYr.tamu-2B* were present in this HWW panel. Using GWAS, we identified 31 SNPs associated with seedling response and 79 SNPs associated with adult plant stage response. Markers linked to *Yr15* and *Yr17* were also among the significant GWAS associations. Furthermore, we found 31 SNPs that were consistently associated with stripe rust response across multiple environments/tests and could be useful for breeding for durable stripe rust resistance. Comparative mapping of significant SNPs identified in this study with previously characterized *Yr* genes/loci indicated that *Yr5/YrSP*, *Yr10*, *Yr15*, *Yr26*, *Yr53*, *Yr62*, *Yr68*, *Yr84*, and *Yr85* are likely present in this HWW panel. Notably, 40 out of 110 SNPs (36%) have not been previously reported. Additionally, the identified 32 wheat genotypes with a high number of stripe rust resistance alleles (79–85) could confer durable stripe rust resistance.

## Supplementary Information

Below is the link to the electronic supplementary material.

Supplementary file1 (DOCX 3410 KB)

Supplementary file2 (XLSX 1442 KB)

## Data Availability

All data generated or analyzed during this study are included in this published article and its supplementary information files submitted with this manuscript. The MRASeq SNP data for 459 hard winter wheat genotypes are available at figshare.com/s/5bba9c3582262a90cc1b.
