# Supplementary material for: Genome-wide association mapping for the identification of stripe rust resistance loci in US hard winter wheat
Source: Theor Appl Genet. 2025 Mar 10;138(4):67. doi: 10.1007/s00122-025-04858-3 (PMC11893644; doi:10.1007/s00122-025-04858-3)
Supplement: Supplementary file 1 — Supplementary file1 (DOCX 3410 KB) [file 122_2025_4858_MOESM1_ESM.docx]

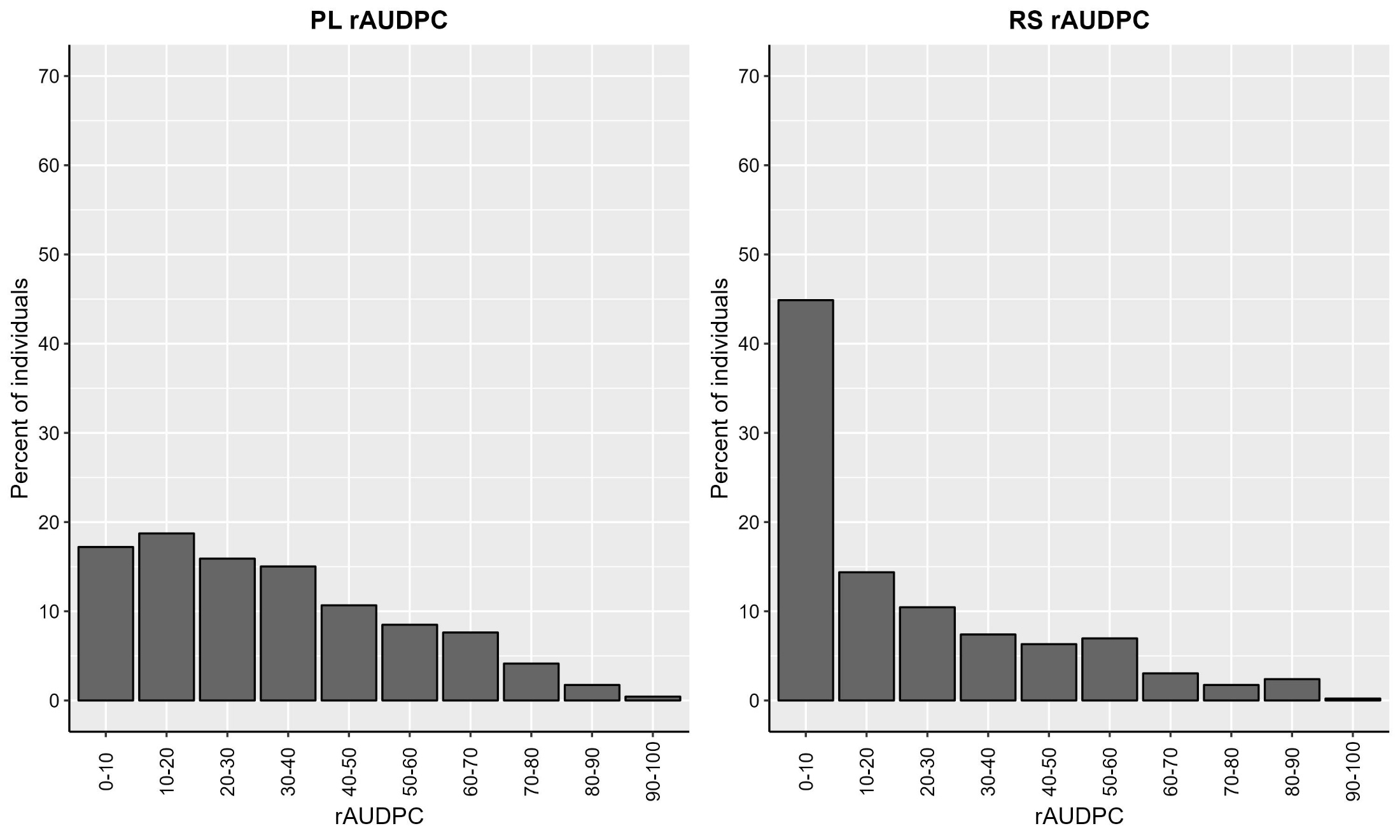


**Fig. S1** Distributions of stripe rust relative area under the disease progress curve (rAUDPC) across field environments. PL = Pullman, WA; RS = Rossville, KS.


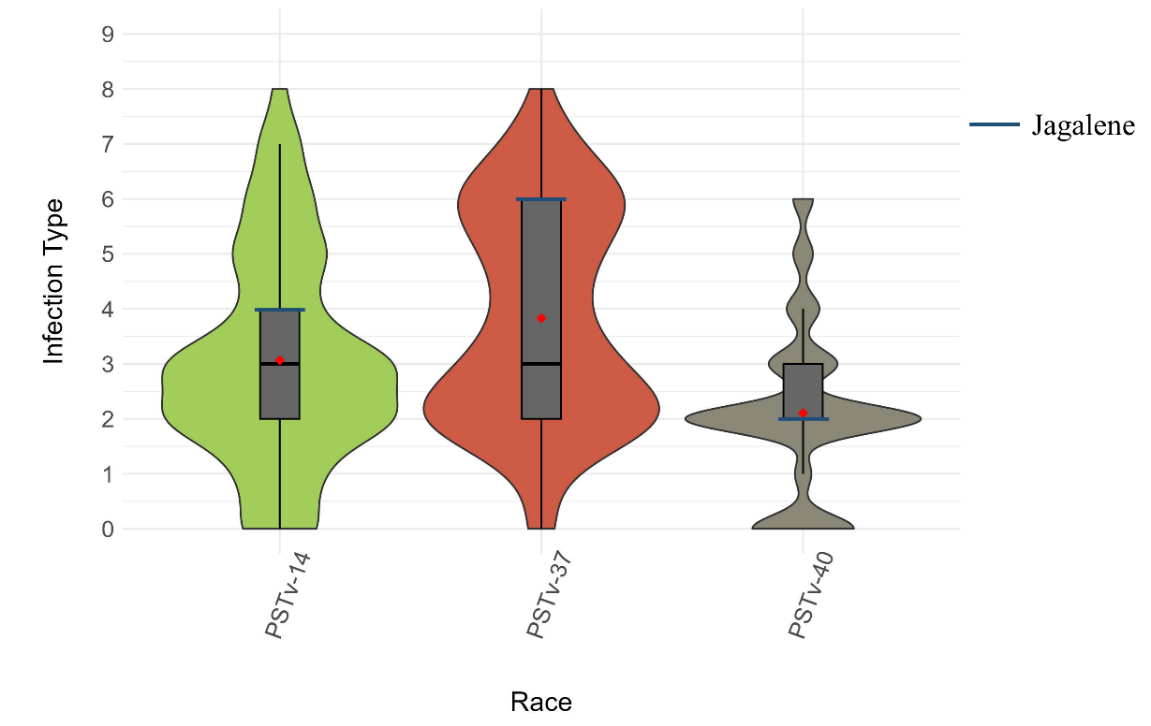


**Fig. S2** Distribution of infection types of 151 NRPN and SRPN (n=151) genotypes against three *Puccinia striiformis f. sp. tritici* races at the adult plant stage in the greenhouse. The black bold horizontal lines in the box plots denote the medians, and the red diamonds represent the means. The blue horizontal lines correspond to stripe rust responses of the susceptible check Jagalene.

**
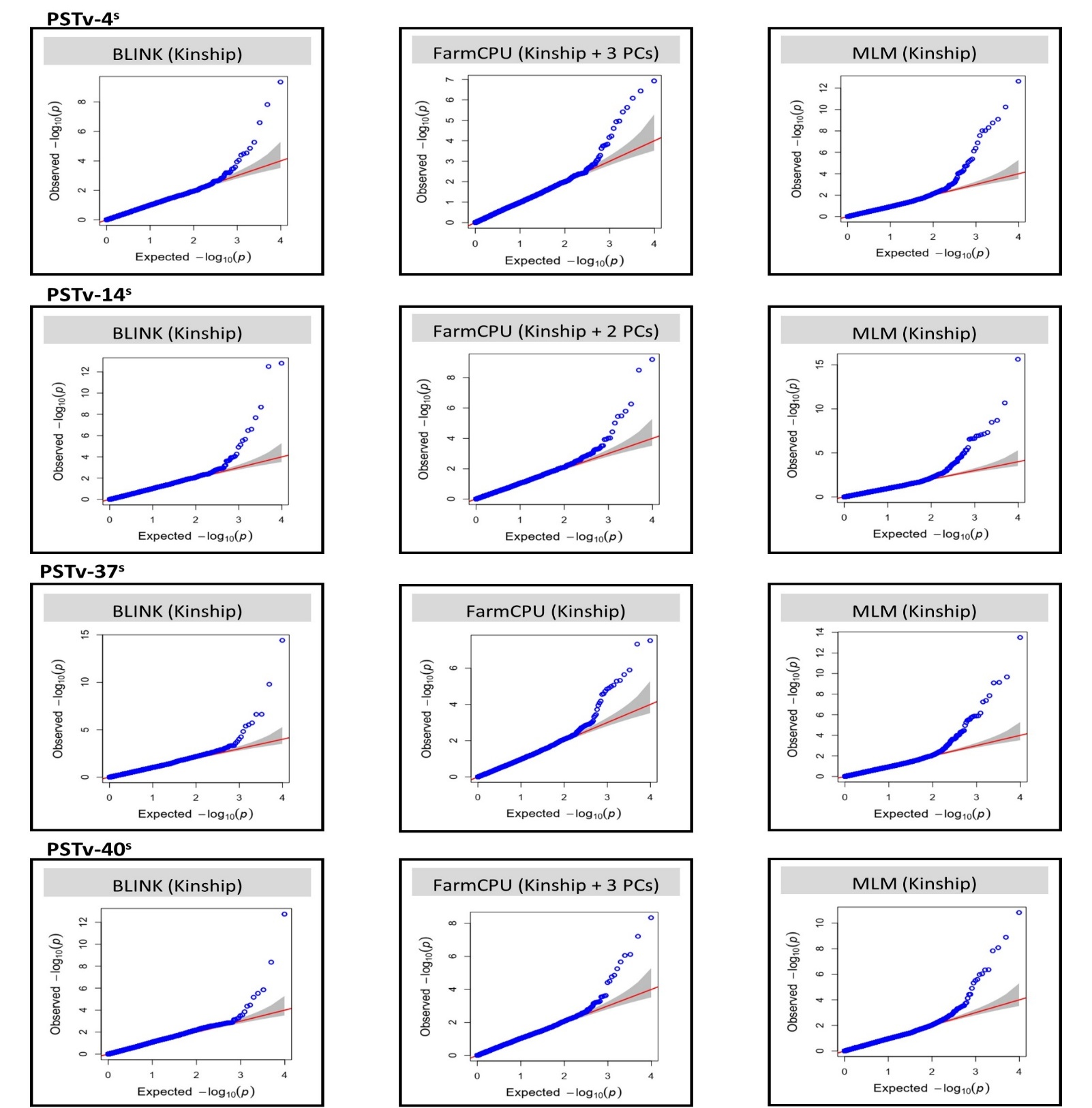
Fig. S3** Quantile-quantile (Q-Q) plots comparing the expected -log10 (*P*) values versus the observed -log10 (*P*) values for different association mapping models, including BLINK, FarmCPU, and MLM, across various traits. ^s^ = Infection type at the seedling stage; ^a^ = Infection type at the adult plant stage. IT = Infection type; DS = Disease severity (%); CH = Chickasha, OK; MV 1 = first disease rating at Mount Vernon, WA; MV 2 = second disease rating at Mount Vernon, WA; PL 1 = first disease rating at Pullman, WA; PL 2 = second disease rating at Pullman, WA; PL 3 = third disease rating at Pullman, WA; PL rAUDPC = relative area under disease progress curve calculated based on Pullman DS data; RS 1 = first disease rating at Rossville, KS; RS 2 = second disease rating at Rossville, KS; RS rAUDPC = relative area under disease progress curve calculated based on Rossville DS data; BLUE = Multi-environment best linear unbiased estimates.


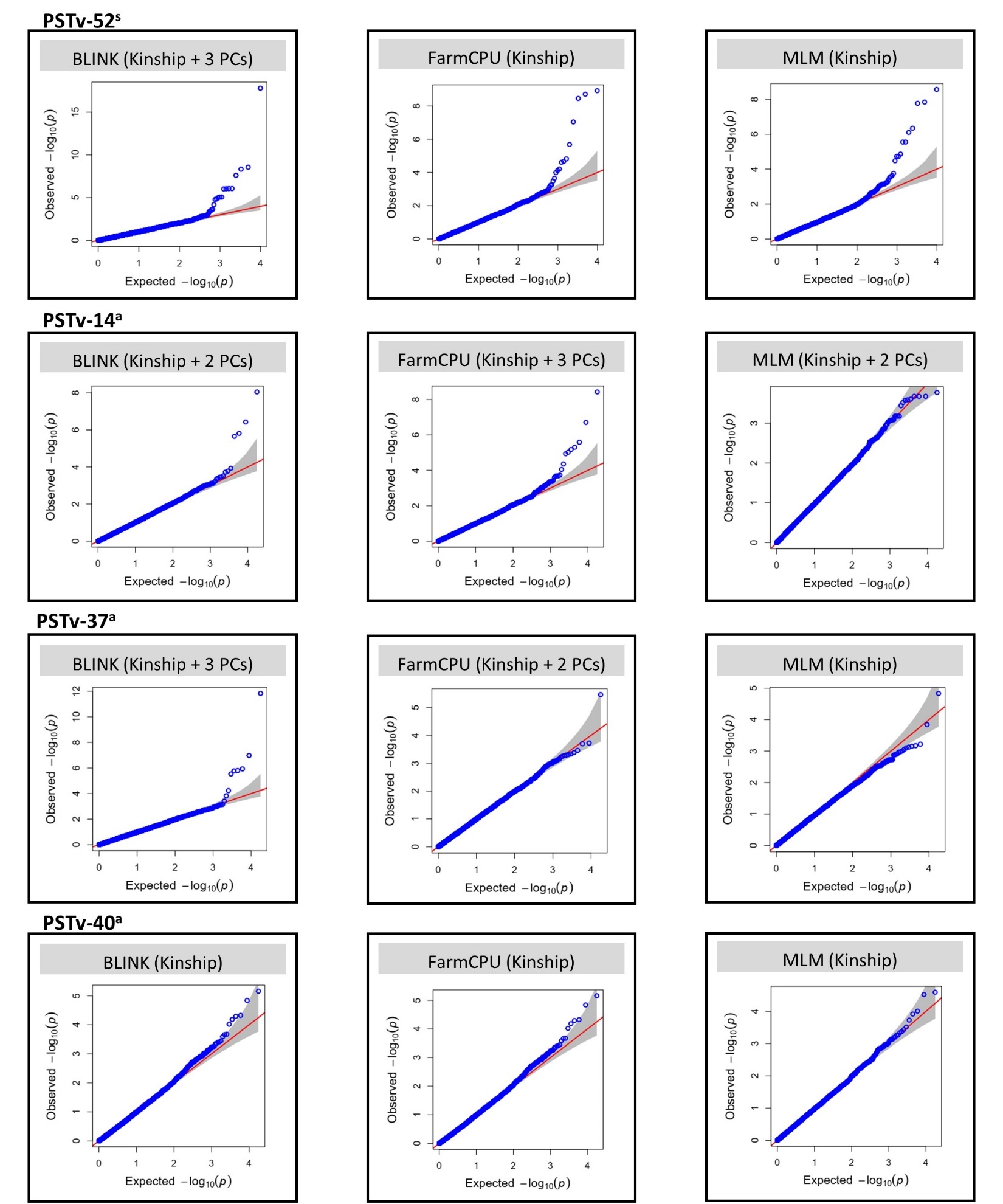
**Fig. S3** (continued)


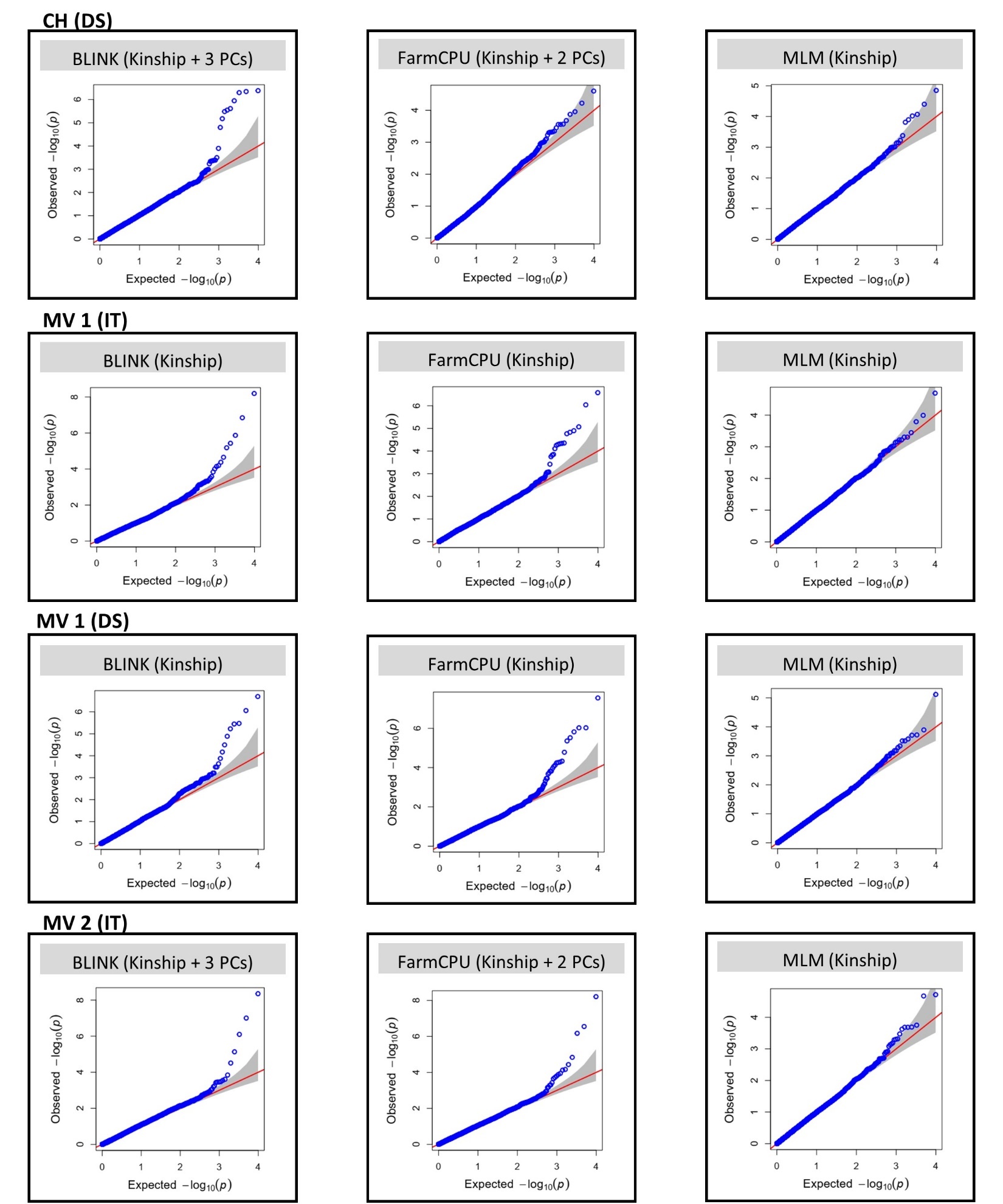
**Fig. S3** (continued)


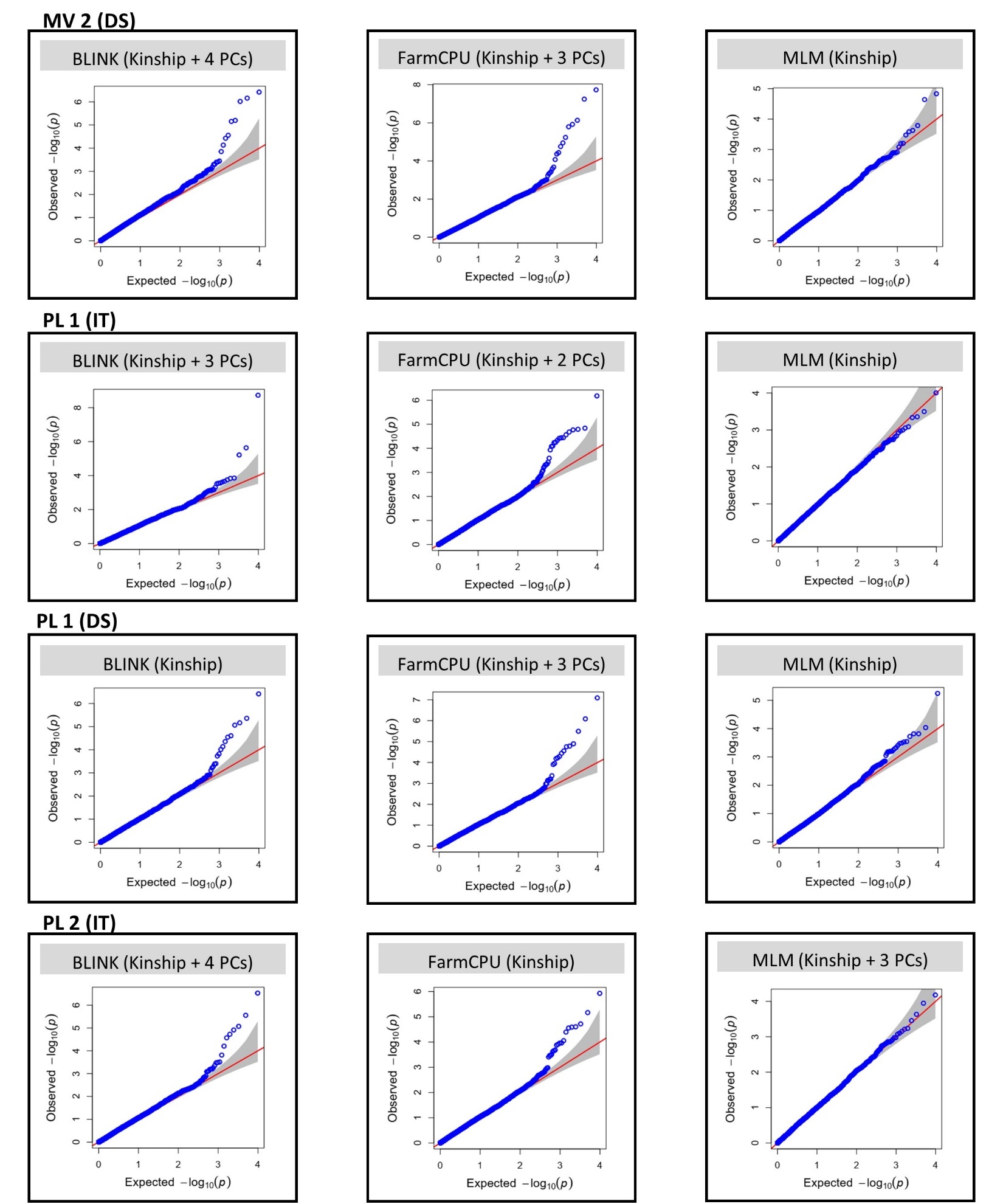
**Fig. S3** (continued)

**
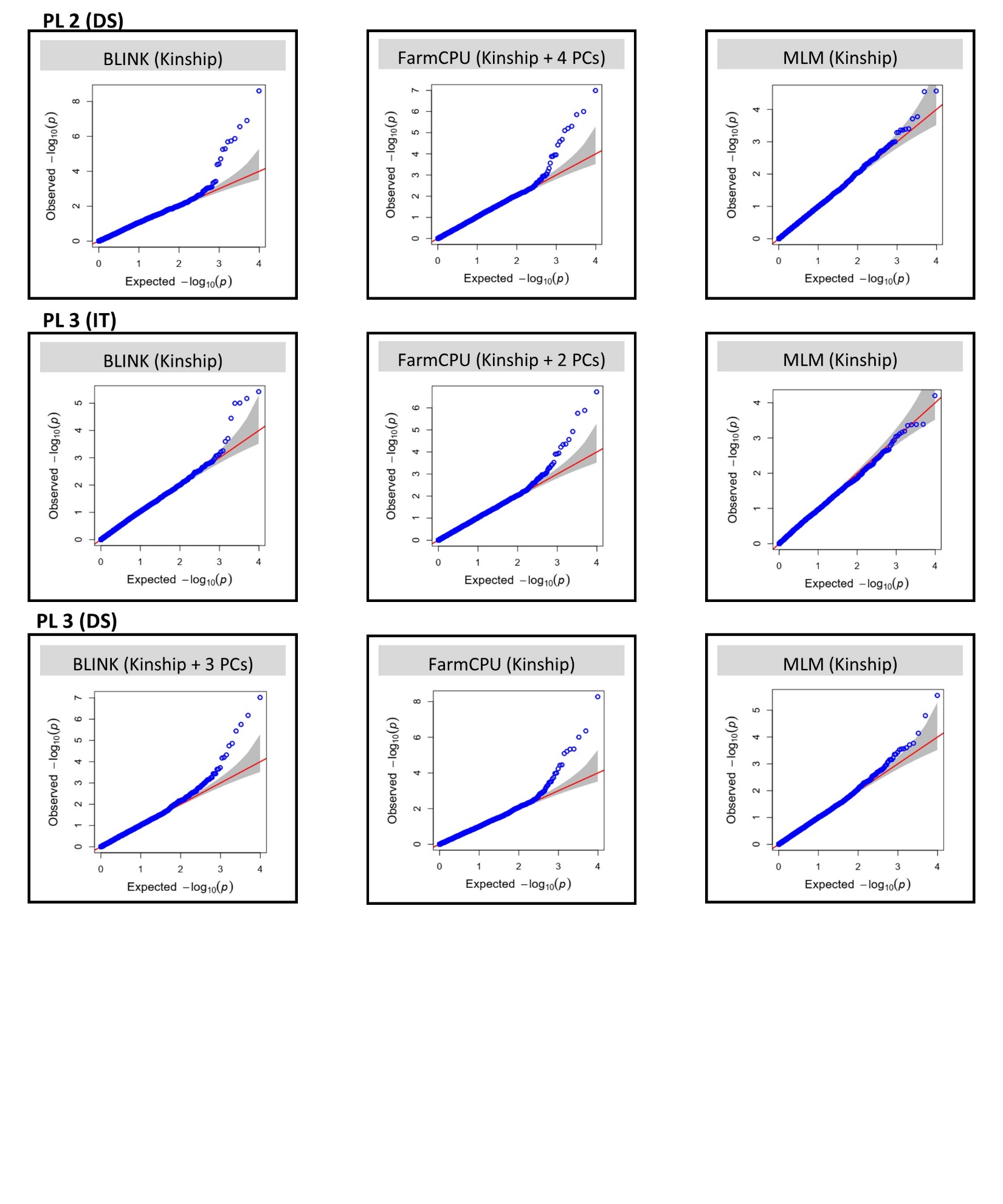
Fig. S3** (continued)

**
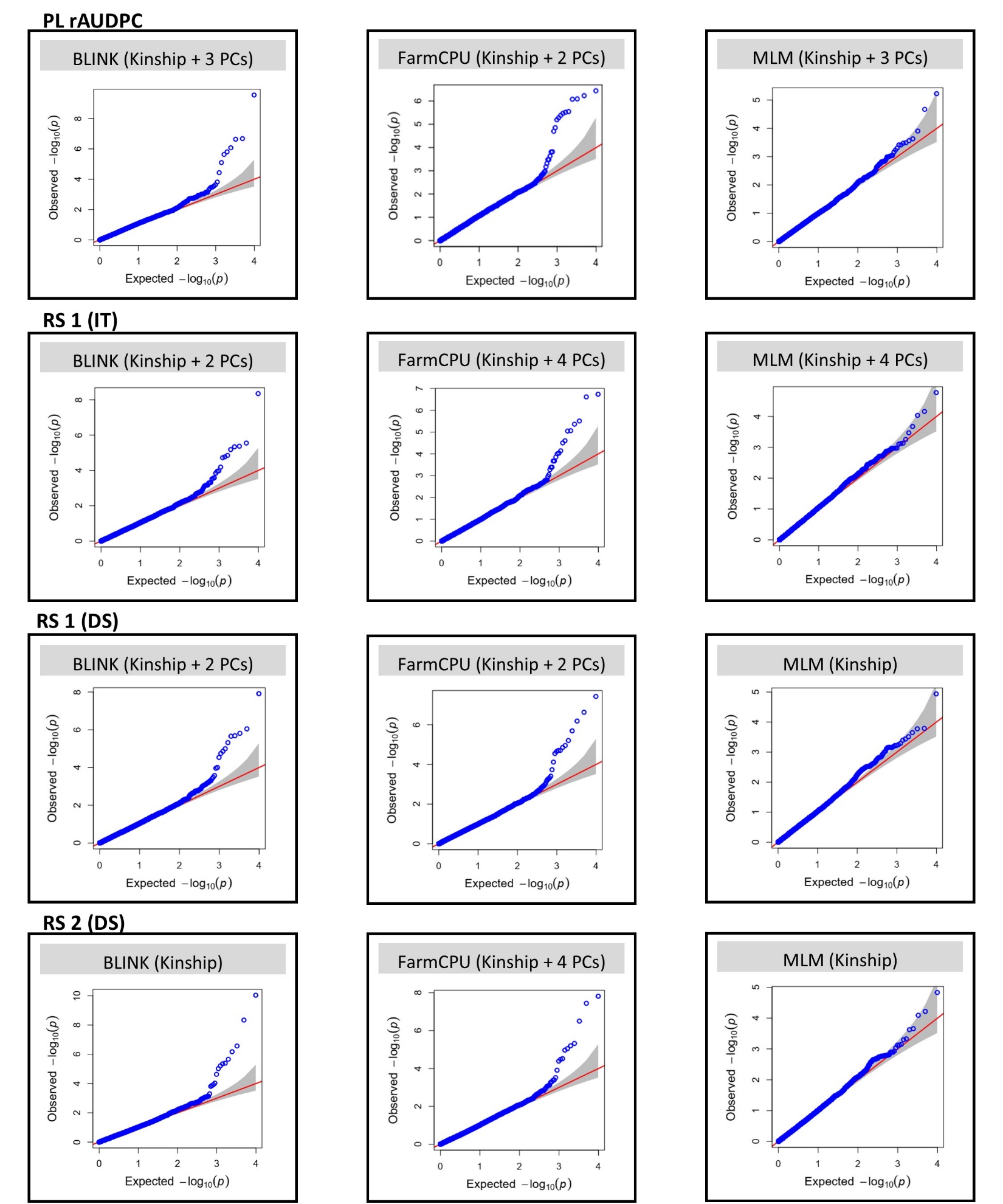
Fig. S3** (continued)

**
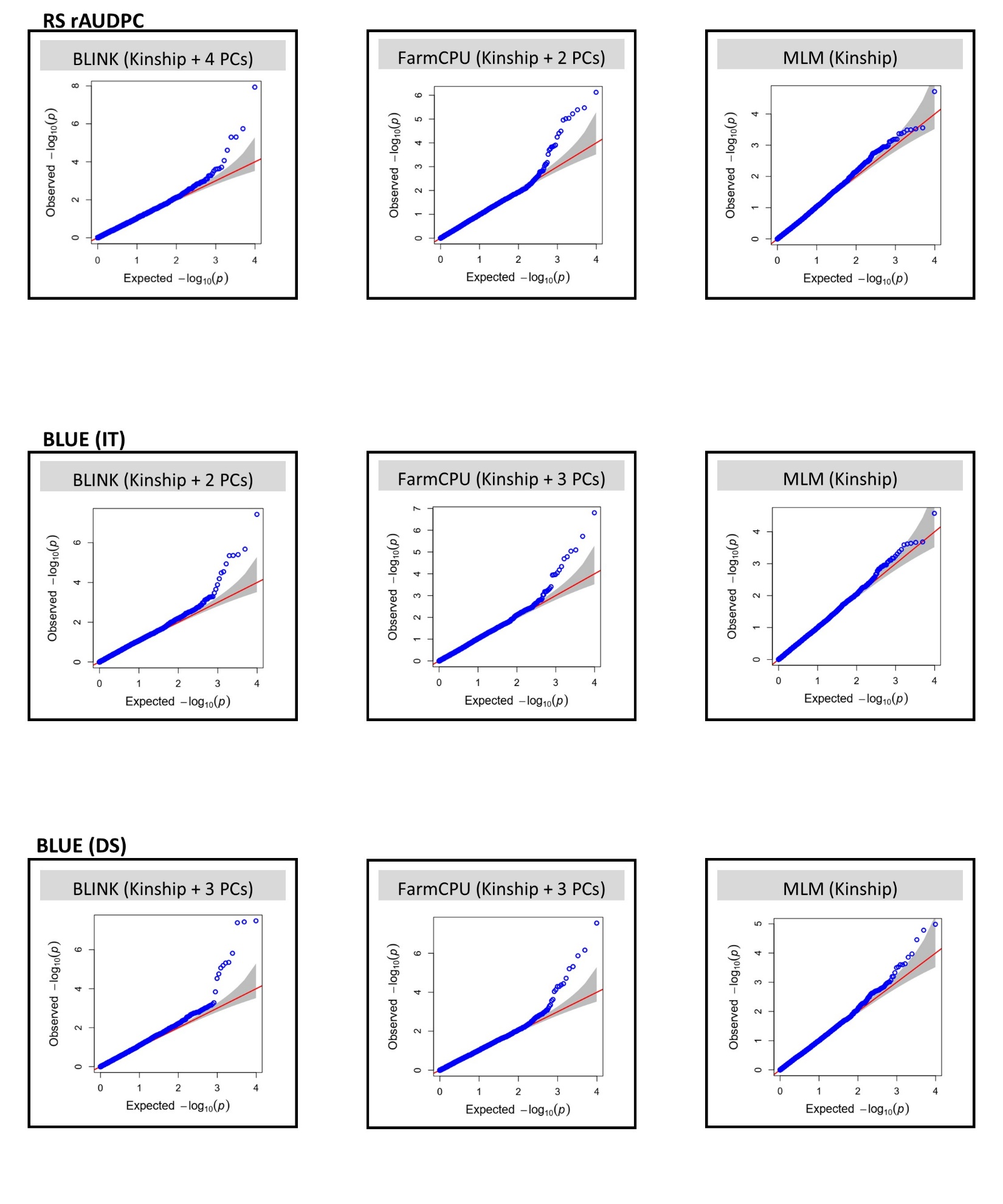
Fig. S3** (continued)
